# Reorientation of INO80 on hexasomes reveals basis for mechanistic versatility

**DOI:** 10.1126/science.adf4197

**Published:** 2023-06-29

**Authors:** Hao Wu, Elise N. Muñoz, Laura J. Hsieh, Un Seng Chio, Muryam A. Gourdet, Geeta J. Narlikar, Yifan Cheng

**Affiliations:** 1 Department of Biochemistry and Biophysics, University of California San Francisco, San Francisco, CA 94158, USA; 2 Tetrad Graduate Program, University of California San Francisco, San Francisco, CA 94158, USA; 3 Howard Hughes Medical Institute, University of California San Francisco, San Francisco, CA 94158, USA

## Abstract

Unlike other chromatin remodelers, INO80 preferentially mobilizes hexasomes, which can form during transcription. Why INO80 prefers hexasomes over nucleosomes remains unclear. Here, we report structures of *S. cerevisiae* INO80 bound to a hexasome or a nucleosome. INO80 binds the two substrates in substantially different orientations. On a hexasome, INO80 places its ATPase subunit, Ino80, at superhelical location (SHL)-2, across from SHL-6/−7 as previously seen on nucleosomes. Our results suggest that INO80 action on hexasomes resembles action by other remodelers on nucleosomes, such that Ino80 is maximally active near SHL-2. The SHL-2 position also plays a critical role for nucleosome remodeling by INO80. Overall, the mechanistic adaptations used by INO80 for preferential hexasome sliding imply that sub-nucleosomal particles play considerable regulatory roles.

## One-Sentence Summary

Structural studies reveal that INO80 binds hexasomes and nucleosomes in dramatically different orientations, providing mechanistic insight into how hexasomes are regulated.

In eukaryotes, central nuclear processes such as gene expression, DNA replication and DNA repair are coordinated with dynamic changes in chromatin states (1-3). ATP-dependent chromatin remodeling enzymes play essential roles in catalyzing such changes. These enzymes are broadly categorized into four major families: SWI/SNF, ISWI, CHD, and INO80 (4, 5). Each of them contains a core remodeling ATPase subunit and several auxiliary subunits that regulate the core ATPase. It has typically been presumed that the preferred substrate of these enzymes is a nucleosome, the smallest unit of chromatin, containing ~147 bp of DNA wrapped around an octamer of histone proteins (6). Consistent with this assumption, between them, these four classes slide the histone octamer, exchange histone variants, and transfer entire octamers (5, 7).

The INO80 complex has been shown to play roles in regulating transcription, DNA replication and DNA repair (8-11). How INO80’s biochemical activities relate to its diverse biological roles is not well understood. Unlike remodelers from other families, whose core ATPase subunits bind the nucleosome near superhelical location (SHL)-2, Ino80, the core ATPase subunit of the INO80 complex, binds nucleosomes near SHL-6/−7 (fig. S1A)(12-14). It has been speculated that this key difference in nucleosome engagement reflects a fundamentally different remodeling mechanism (15, 16). Indeed, we showed that the preferred substrate of the *S. cerevisiae* INO80 complex is not a nucleosome but a hexasome, which is a sub-nucleosomal particle that lacks a histone H2A-H2B dimer (17). Hexasomes are generated during transcription and may also be formed during DNA replication and repair (18-21). Further, INO80’s activity on nucleosomes is more dependent on flanking DNA length than on hexasomes (17, 22). These results suggested that INO80 has the versatility to act on hexasomes or nucleosomes based on the density of nucleosomes and hexasomes at a given locus. Yet, fundamental mechanistic questions remain. It is not clear how INO80 can act on both nucleosomes and hexasomes, which differ substantially in their structures. Additionally, why INO80 has different flanking DNA length dependencies on hexasomes versus nucleosomes is unclear.

Here, we report cryogenic-electron microscopy (cryo-EM) structures of endogenously purified *S. cerevisiae* INO80 bound to a hexasome and a nucleosome. We find that INO80 binds hexasomes and nucleosomes in opposite orientations, with Ino80 binding near SHL-2 on hexasomes and near −6/−7 on nucleosomes. The location of the Arp8 module suggests how flanking DNA length differentially regulates nucleosome and hexasome sliding. DNA gaps near SHL-2 inhibit sliding of both substrates by INO80. Together, our findings provide mechanistic insights into how INO80 slides both hexasomes and nucleosomes.

## Structures of the INO80-hexasome and -nucleosome complexes

**Fig. 1.**
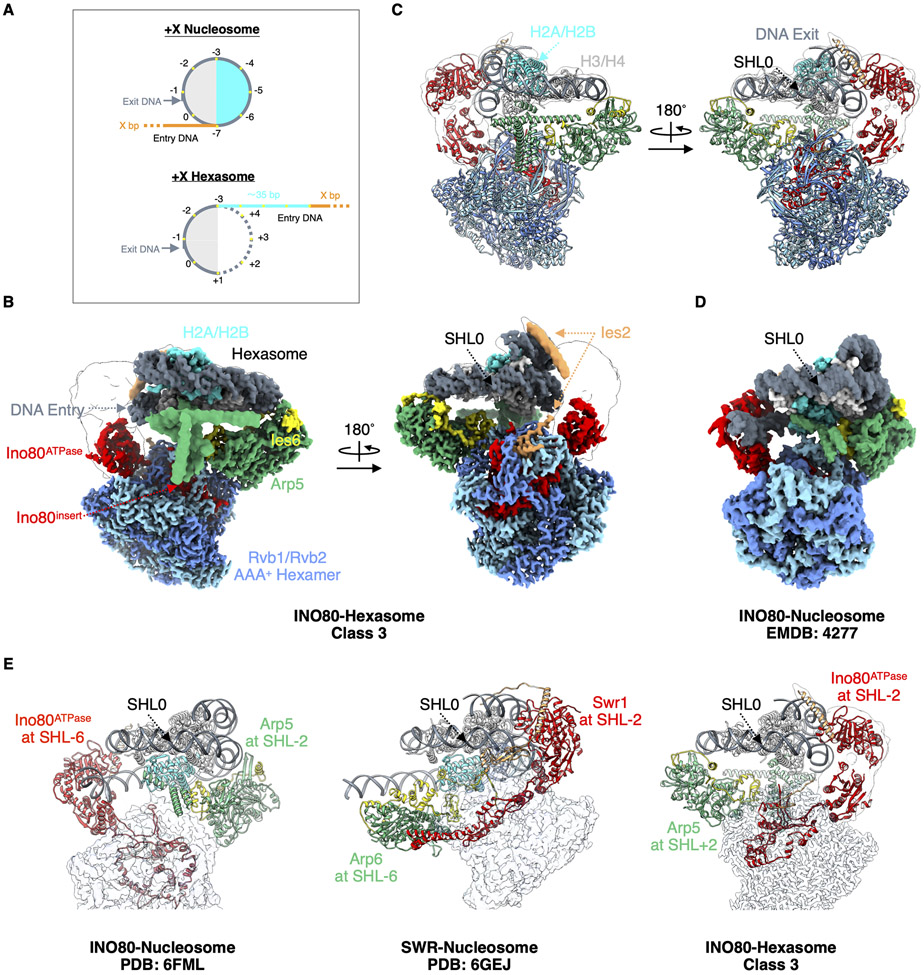
Structure of the INO80-hexasome complex reveals large rotation. (A) Cartoon illustration of a +X Nucleosome and a +X Hexasome. H2A-H2B dimer proximal to the flanking DNA (entry side dimer): cyan; H3-H4: light gray; 601 DNA: dark gray; flanking DNA: orange; additional free (unwrapped) DNA: cyan; super helical locations: yellow dots; DNA from the bottom gyre: dotted line. (B) Two different views of cryo-EM density map of the INO80-hexasome complex (class 3). (C) Atomic model of the INO80-hexasome complex (class 3), viewed in the same orientation as the map is viewed in (B). (D) Cryo-EM density map of *Chaetomium thermophilum* INO80-nucleosome complex (EMDB: 4277 (14)) displayed with its nucleosome dyad and H3-H4 tetramer aligned with that of the hexasome in the right panel of (B). Note that INO80 on a hexasome rotates ~180° from where it sits on a nucleosome when keeping the nucleosome/hexasome dyad and H3-H4 aligned. (E) Structural comparisons of INO80-nucleosome complex (left), SWR-nucleosome complex (middle) and INO80-hexasome complex (right), with nucleosome/hexasome dyad and H3-H4 aligned.

To visualize how INO80 binds to a hexasome or a nucleosome, we prepared hexasomes and nucleosomes on the same DNA templates containing the 147 bp 601 nucleosome positioning sequence with 80 bp of additional DNA as described previously (+80H and +80N, with definition explained in Fig. 1A, and fig. S1, A and B, and Supplementary Text) (17, 23, 24). Complexes were formed by mixing hexasomes or nucleosomes with endogenously purified *S. cerevisiae* INO80 without adding nucleotide (fig. S1, C to H).

We determined cryo-EM structures of the INO80-hexasome complex in three different conformational snapshots (Fig. 1, B and C, and figs. S2 to S6). The overall shape of INO80 is similar within these structures and also to previously published structures of the nucleosome in complex with human (12) and *Chaetomium thermophilum* (14) INO80. We group subunits of the INO80 complex into four modules: Rvb module (Rvb1/Rvb2), Arp8 module (Arp8/Arp4/Actin/Ies4 and Taf14), Ino80 module (Ino80/Ies2) and Arp5 module (Arp5/Ies6). The Ino80 protein consists of three major regions: the N-terminal domain (NTD), the HSA region (Ino80^HSA^) and the ATPase domain (Ino80^ATPase^). Detailed descriptions of these modules in our structures are in the Supplementary Text.

While the INO80 architecture appears similar to that in the INO80-nucleosome structures, a major difference is that it is rotated ~180° on a hexasome compared to a nucleosome (Fig. 1, B to E). We identified two primary interactions between INO80 and the hexasome: Ino80^ATPase^ binds the hexasome near SHL-3 (class 1), −2.5 (class 2) and −2 (class 3), and the Arp5/Ies6 module binds near SHL+1, +1.5 and +2 (fig. S6, A and B) respectively. Class 3 is the predominant INO80-hexasome class. All Ino80^ATPase^ locations on hexasomes are different than on nucleosomes, which are near SHL-6 or SHL-7 (12-14). However, the Ino80 orientation on hexasomes is consistent with structures of other major chromatin remodelers on nucleosomes such as *S. cerevisiae* ISW1 (25-27), Chd1 (28-30), RSC (31-33), Snf2 (34) and in particular the SWR1 complex (35), which is from the same sub-family as the INO80 complex. In these structures the ATPase domains interact with nucleosomes near either SHL+2 or SHL-2 (Fig. 1E).

**Fig. 2.**
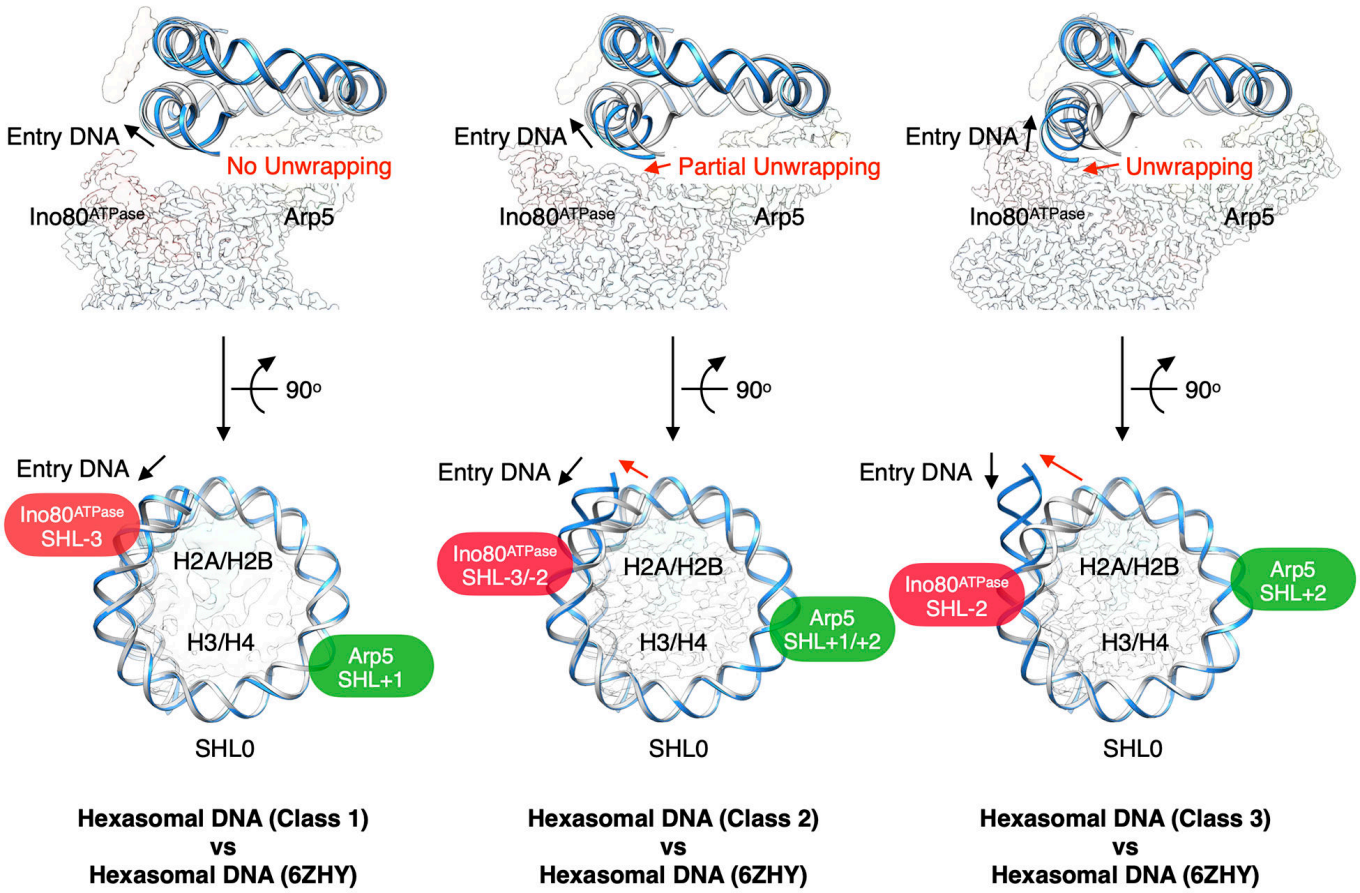
Conformational snapshots of INO80-hexasome complexes. Comparison of DNA from each INO80-hexasome class (blue) with DNA from an unbound hexasome (PDB: 6ZHY, gray), showing degree of DNA unwrapping (upper row) and binding locations of Ino80^ATPase^ and Arp5 (bottom row).

Loss of an H2A-H2B dimer in a hexasome causes an additional ~35 bp of DNA to unwrap from the histone core (free DNA) (Fig. 1A, and fig. S1B). Comparison of our hexasome structures with an unbound hexasome (PDB: 6ZHY, (36)) reveals different levels of further DNA unwrapping. In class 1, the hexasome is almost identical with the unbound hexasome, without detectable additional DNA unwrapping. The level of DNA unwrapping increases as the Ino80^ATPase^ binding position changes from SHL-3 (class 1) to SHL-2 (class 3) (Fig. 2, and fig. S6C).

For comparison, we also determined structures of *S. cerevisiae* INO80 bound to a nucleosome and captured two conformational snapshots (class 1 and 2) from the same dataset (figs. S7 to S9, Supplementary Text). Ino80^ATPase^ in class 1 is located near SHL-7, similar to its location in the human INO80-nucleosome structure (12), while in class 2, it binds near SHL-6, similar to the *C. thermophilum* structure (14) (fig. S9, A and C). The Arp5/Ies6 module interacts with the nucleosome near SHL-3 and SHL-2 (fig. S9D), respectively. These observations are also consistent with previous findings showing that nucleosomal DNA between SHL-7 and −6 is protected by INO80 (13).

## The SHL-2 position plays a critical role in nucleosome and hexasome sliding

**Fig. 3.**
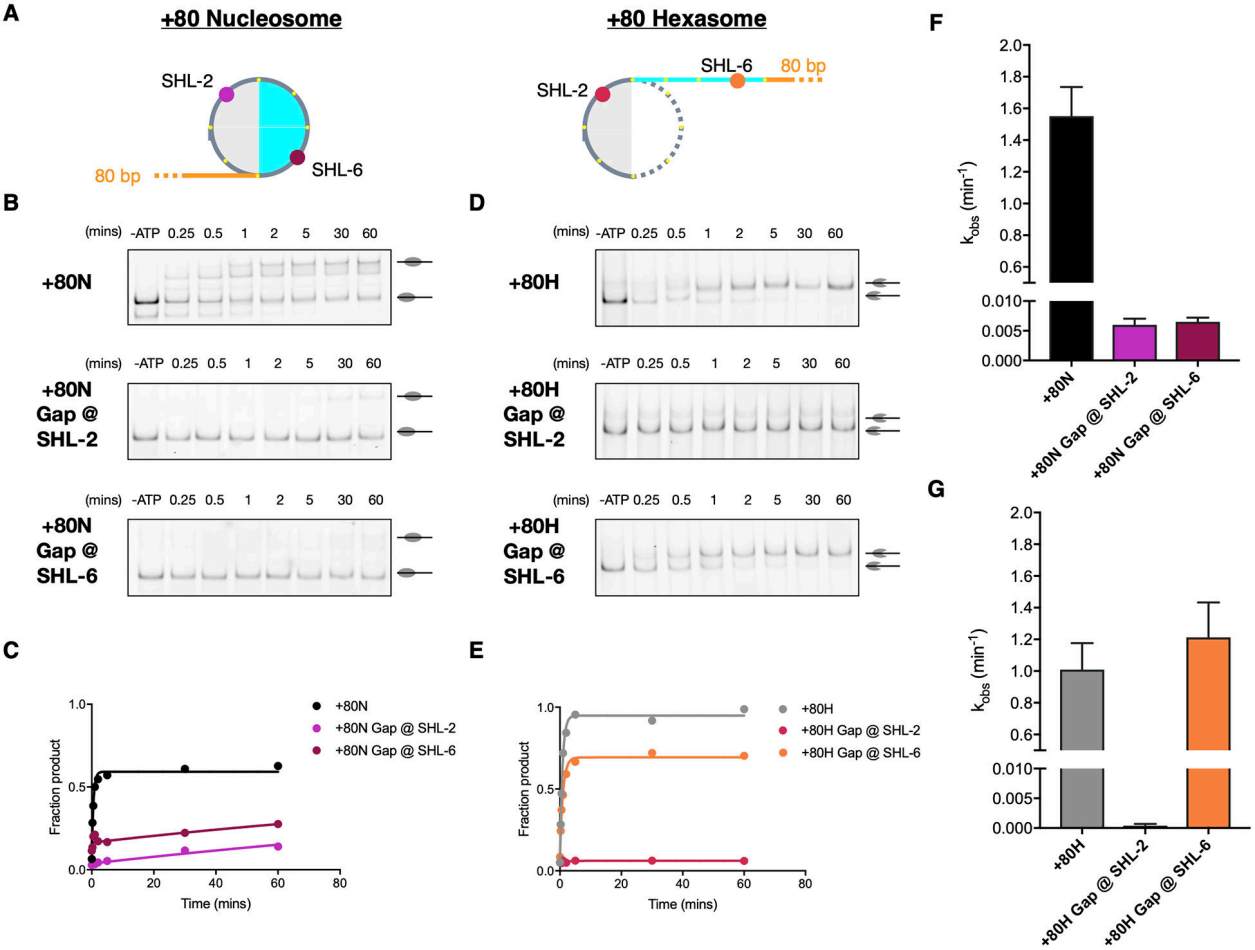
Inhibition of DNA translocation at specific SHL sites influence nucleosome and hexasome sliding by INO80. (A) Cartoon illustration of a +80 Nucleosome (left) and a +80 Hexasome (right) with approximate locations of site-specific single base gaps indicated. Colors are the same as in Fig. 1A. (B-C) Example gels and time courses of native gel-based remodeling assays of WT INO80 on +80 nucleosomes with no gap, gap near SHL-2, and gap near SHL-6. (D-E) Example gels and time courses of native gel-based remodeling assays of WT INO80 on +80 hexasomes with no gap, gap near SHL-2, and gap near SHL-6. (F-G) Average observed rate constants of INO80 sliding activity. k_obs_ (min^−1^): +80N: 1.551 ± 0.1846; +80N Gap @ SHL-2: 0.005995 ± 0.001054; +80N Gap @ SHL-6: 0.006497 ± 0.0007117; +80H: 1.01 ± 0.1668; +80H Gap @ SHL-2: 0.000379 ± 0.0002849; +80H Gap @ SHL-6: 1.213 ± 0.2209. Data represent the mean ± SEM for three technical replicates performed under single-turnover conditions with saturating enzyme and ATP.

We observe that Ino80^ATPase^ engages the hexasome predominantly near SHL −2. These results raise the possibility that Ino80^ATPase^ acts near SHL-2 when sliding hexasomes. In contrast, consistent with prior findings (12, 14), we observe that Ino80^ATPase^ engages the nucleosome near two positions, SHL-7 and −6. Also as previously proposed, our findings are consistent with the possibility that Ino80^ATPase^ acts near SHL-6 when sliding nucleosomes (13). A commonly used assay to identify the DNA location from where the ATPase domain of a remodeler acts to translocate DNA is to place a single nucleotide gap at the proposed site of action and test if the gap inhibits DNA translocation (37-39). Therefore, to directly test the importance of the SHL-6 and SHL-2 locations, we assembled nucleosomes and hexasomes with single base gaps near SHL-2 or SHL-6 and measured INO80 activity using a gel-based sliding assay (Fig. 3A).

We found that a gap at SHL-6 inhibits INO80’s sliding activity on nucleosomes by ~ 200-fold but so did a gap at SHL-2 (Fig. 3, B to G). In contrast, a gap at SHL-6 did not inhibit INO80’s sliding activity on hexasomes, but a gap at SHL-2 slowed hexasomes sliding by ~ 2000-fold (Fig. 3, B to G). These results are consistent with Ino80^ATPase^ acting near SHL-2 when sliding hexasomes and raise new questions about why both the SHL-2 and SHL-6 locations are critical for nucleosome sliding by INO80. We describe possible explanations in the Discussion.

## The role of the Arp8 module in flanking DNA length dependence

**Fig. 4.**
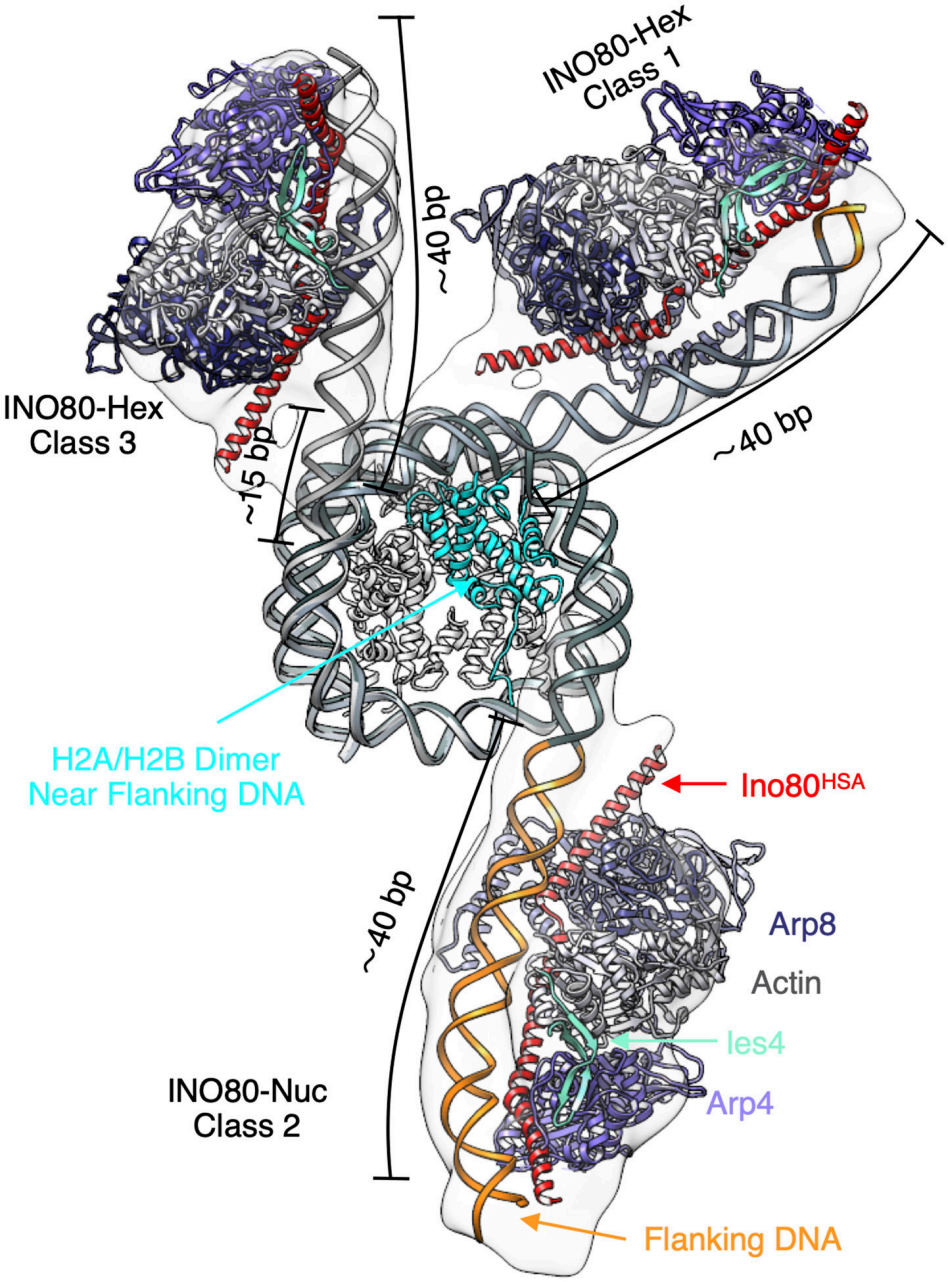
The Arp8 module engages different regions of DNA in nucleosomes versus hexasomes. Overlay of atomic models of the hexasome (class 1 and class 3) and the nucleosome (class 2) with the Arp8 module (PDB: 8A5O), aligned by the H3-H4 tetramer.

*S. cerevisiae* INO80 slides +40 nucleosomes ~100-fold slower than +80 nucleosomes (17, 22). However, sliding hexasomes is less flanking DNA dependent. Our structures suggest that the Arp8 module requires ~40 bp of DNA for appropriate engagement. In class 1 of the INO80-hexasome structure, Arp8 engages with the ~35 bp of DNA unwrapped from removal of the H2A-H2B dimer and an additional ~5 bp of flanking DNA. In class 3 of the INO80-hexasome structure, the Arp8 module engages entirely with ~40 bp of unwrapped DNA that now includes additional DNA unwrapped relative to the unbound hexasome (Fig. 4). In contrast, in class 2 of the INO80-nucleosome structure, the Arp8 module engages entirely with flanking DNA consistent with previous findings (40) (Fig. 4). Our structural data with hexasomes along with the previous data with nucleosomes suggest that 40 bp may be the minimum amount of DNA needed for the Arp8 module to bind and that proper Arp8 module engagement is essential for maximal remodeling activity (40).

## Altered interactions by the Arp5 module

To understand why Ino80 may not bind a nucleosome directly near SHL-2, we compared interactions made by Arp5/Ies6 in hexasomes versus nucleosomes (Supplementary Text). When INO80 binds to a hexasome, the Arp5/Ies6 regions used in the context of a nucleosome are repurposed for different interactions. Modeling the missing H2A-H2B dimer into our INO80-hexasome structure reveals steric clashes of the Arp5 module with the entry side proximal H2A-H2B dimer and with part of the DNA that wraps around the H2A-H2B dimer near SHL-2 (fig. S11). These clashes could be avoided if the H2A-H2B dimer is sufficiently dislodged. To test for this possibility, we inhibited dimer dislodgement by introducing a site-specific disulfide crosslink between the two H2A molecules (N38C) (41) or promoted dimer dislodgement by using an H2A mutant (R81A) that destabilizes the H2A-H2B/H3-H4 interface (42) (fig. S12, A, B and H). The disulfide crosslink did not inhibit nucleosome sliding while the H2A mutant did not promote nucleosome sliding (fig. S12, C to G), indicating that complete dimer dislodgement is not necessary for INO80-mediated nucleosome sliding. In the absence of dimer dislodgement, another way to avoid these clashes could be by substantial rearrangement of the Arp5 module together with subtle rearrangements of the H2A-H2B dimer (fig. S9E).

## DISCUSSION

### Implications of the INO80-hexasome structure for nucleosome sliding by INO80

**Fig. 5.**
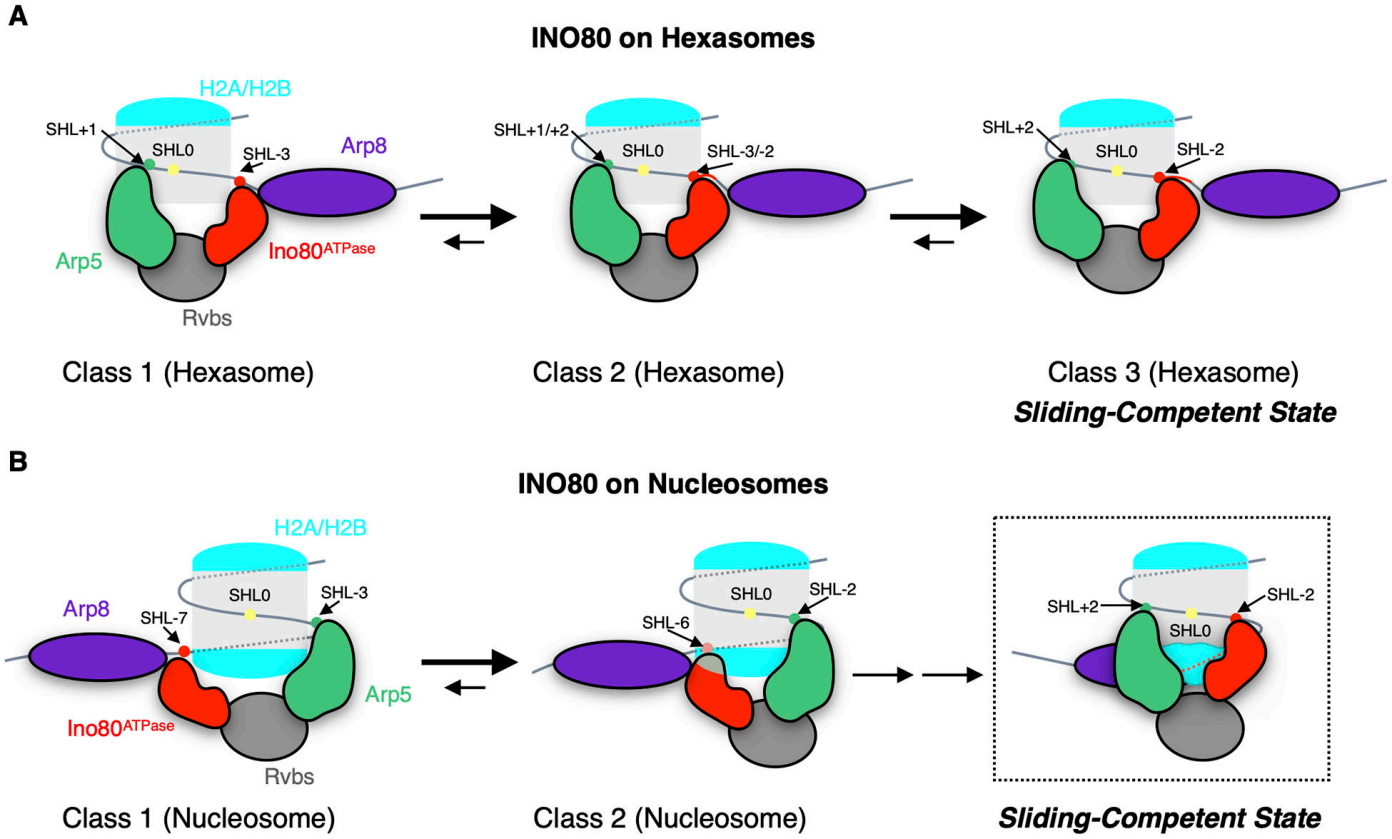
Model of INO80-induced hexasome and nucleosome sliding. (A) Hexasome sliding: the Ino80^ATPase^ samples different positions between SHL-3 and SHL-2 but binds predominantly near SHL-2. The INO80 complex becomes sliding-competent when Ino80^ATPase^ engages near SHL-2. (B) Nucleosome sliding: INO80 initially binds with Ino80^ATPase^ at SHL-7 or −6. Upon ATP-hydrolysis, Ino80^ATPase^ moves toward SHL-2 where INO80 becomes sliding-competent.

The major conformation of the INO80-hexasome complex (class 3) has Ino80^ATPase^ near SHL-2 and approximately ~15bp of unwrapped DNA from the entry site in addition to the ~35bp of DNA that is unwrapped from removal of an H2A-H2B dimer. The placement of Ino80^ATPase^ near SHL-2 is consistent with how the ATPase subunits of other remodelers bind the nucleosome. Together with our prior finding that hexasomes are remodeled faster than nucleosomes, these results suggest that the class 3 structure represents the sliding-competent conformation of INO80 on hexasomes (Fig. 5A, and fig. S13A). In contrast, the states of INO80 bound to a nucleosome have Ino80^ATPase^ bound near either SHL-6 or −7 consistent with previous findings. These differences raise the question of whether the INO80-nucleosome structures represent sliding-competent conformations or whether a rearrangement of Ino80^ATPase^ to SHL-2 is necessary to achieve efficient nucleosome sliding.

Previous crosslinking studies have shown that detachment of nucleosomal DNA from H2A-H2B close to the entry site occurs during INO80 remodeling (13). Our data show that progressively more DNA is unwrapped as Ino80^ATPase^ binds closer to SHL-2 on hexasomes (Fig. 2, and fig. S6C). Together these results suggest that DNA unwrapping is coupled to Ino80^ATPase^ accessing its most sliding-competent state. Foot-printing studies have shown that while binding of INO80 to nucleosomes mainly protects nucleosomal DNA from SHL-5 to SHL-6 and near SHL-3, there is modest but detectable protection near SHL-2 (13). Nicks and gaps between SHL-7 and SHL −2 have been shown to inhibit nucleosome sliding to different extents (13, 43). Here we show that site-specific gaps near SHL-2 or SHL-6 substantially inhibit INO80’s sliding of nucleosomes (by ~200 fold). DNA gaps are commonly used to identify the site of action of the ATPase domain of remodelers (37-39). We therefore speculate that INO80 initially binds the nucleosome with Ino80^ATPase^ near SHL-6/−7, and this is followed by an ATP-dependent rotation around the nucleosome to position Ino80^ATPase^ near SHL-2 from where Ino80^ATPase^ then translocates nucleosomal DNA (Fig. 5B, and fig. S14A). A gap at SHL-6 would then inhibit ATP-dependent movement of Ino80^ATPase^ on the nucleosome while the gap at SHL-2 would inhibit translocation of nucleosomal DNA by INO80 relative to the histone octamer (fig. S14). Single-molecule FRET studies have identified an ATP-dependent pause phase prior to ATP-dependent nucleosome sliding (22). The pause could represent the reorientation of Ino80^ATPase^ from SHL-6/−7 towards SHL-2 and add a step that slows remodeling of nucleosomes compared to hexasomes. Simply placing the INO80 complex as is on nucleosomes with the Ino80^ATPase^ near SHL-2 results in steric clashes of the Arp5 module with the nucleosome (fig. S11). While partial H2A-H2B dimer dislodgment, as previously proposed, could avoid such clashes (17), our biochemical data here indicate that dimer dislodgement is not essential for nucleosome sliding by INO80 (fig. S12). Thus more structural studies are needed to understand how INO80 might rotate around a nucleosome.

Alternatively, a gap near SHL-2 may affect the action of the Arp5 module. For such a scenario we speculate that Ino80^ATPase^ translocates DNA near SHL-6 and effective translocation also requires action of the Arp5 module near SHL-2 as previously proposed (12, 14). A gap at SHL-6 would then inhibit translocation of nucleosomal DNA by Ino80^ATPase^ and a gap at SHL-2 would inhibit productive engagement of the Arp5 module (fig. S15).

Clearly distinguishing between these two models will require substantial additional structural analysis of INO80 remodeling intermediates on nucleosomes.

### Implications for hexasome sliding by INO80

Our structures provide a view into how INO80 engages a hexasome. In the predominant INO80-hexasome structure, Ino80^ATPase^ binds near SHL-2. A site-specific gap at SHL-2 substantially inhibits INO80’s sliding of hexasomes (~2000 fold) while a gap near SHL-6 does not have a major effect. We therefore hypothesize that Ino80^ATPase^ bound at SHL-2 on a hexasome represents the active structure. Compared to the subtle changes at SHL-2 observed when other remodelers bind nucleosomes (16), the 15 bp of unwrapped DNA (up to SHL-2.5) in class 3 substantially loosens histone DNA interactions and thus may allow more ready translocation from SHL-2. We further propose that the new contacts made by the Arp5/Ies6 module with the exposed H3-H4 surface provide an anchor allowing the Ino80 motor to efficiently pump DNA through the hexasome. These findings also explain the differential effects of the Arp5/Ies6 module on hexasome versus nucleosome sliding (17). The location of the Arp8 module is also different on hexasomes than on nucleosomes. On nucleosomes the Arp8 module binds ~ 40 bp entirely on the flanking DNA (Fig. 4). In the most prevalent INO80-hexasome state (class 3), the Arp8 module is bound entirely to the unwrapped DNA, substantially reducing the need to bind flanking DNA (Fig. 4). These different binding modes of the Arp8 module could explain why hexasome sliding by INO80 is less dependent on flanking DNA length compared to nucleosome sliding.

## Supplementary Material

Supplementary Materials
