# Supplementary material for: Reorientation of INO80 on hexasomes reveals basis for mechanistic versatility
Source: Science. Author manuscript; Available in PMC 2023 Sep 5. (PMC10480058; doi:10.1126/science.adf4197)
Supplement: Supplementary Materials [file NIHMS1928128-supplement-Supplementary_Materials.docx]

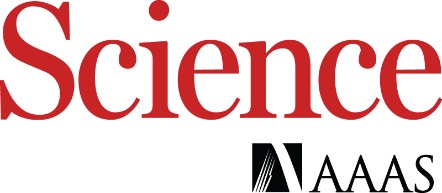


Supplementary Materials for

**Reorientation of INO80 on hexasomes reveals basis for mechanistic versatility**

Hao Wu^1^†, Elise N. Muñoz^1,2^†, Laura J. Hsieh^1^, Un Seng Chio^1^, Muryam A. Gourdet^1,2^*,

Geeta J. Narlikar^1^* and Yifan Cheng^1,3^*

^1^Department of Biochemistry and Biophysics, University of California San Francisco, San Francisco, CA 94158, USA

^2^Tetrad Graduate Program, University of California San Francisco, San Francisco, CA 94158, USA

^3^Howard Hughes Medical Institute, University of California San Francisco, San Francisco, CA 94158, USA

† These authors contributed equally to this work

*Correspondence: [Muryam.Gourdet@ucsf.edu](mailto:Muryam.Gourdet@ucsf.edu), [Geeta.Narlikar@ucsf.edu](mailto:Geeta.Narlikar@ucsf.edu), and [Yifan.Cheng@ucsf.edu](mailto:Yifan.Cheng@ucsf.edu)

**The PDF file includes:**

Materials and Methods

Supplementary Text

Figs. S1 to S15

Table S1 to S2

Materials and Methods

Expression and purification of INO80

A yeast strain containing FLAG-tagged Ino80 (s288c INO80-FLAG) (*11*) was grown in YPD at 30 °C to saturation. INO80 was then purified by FLAG immunoprecipitation based on previously published methods (*17, 22, 44*) with minor modifications. A second 30-minute elution step was added to increase yield.

Preparation of nucleosomes and hexasomes

Recombinant *Xenopus laevis* histones were expressed in *E. coli* and purified as previously described (*45, 46*). DNA was amplified from a plasmid containing a Widom 601 sequence and labeled with a Cy3 fluorophore modified primer (IDT). Large scale PCR was performed, and products were separated on an 8% polyacrylamide gel and cut out. The gel slice containing the DNA was crushed, soaked in 1X TE overnight, and filtered through a 0.45 micron filter. The DNA was ethanol precipitated and dissolved in 1X TE.

Cy3-labeleld DNA containing a single base gap near SHL-2 (25 base pairs from the dyad towards the flanking DNA) and near SHL-6 (58 base pairs from the dyad towards the flanking DNA) were made through annealing and ligating a set of overlapping complementary oligonucleotides, as described previously (*47*). Briefly, single-stranded oligonucleotides spanning the Widom 601 sequence plus 80 base pairs of flanking DNA were designed to have ~20-30 base pairs of overlap with oligonucleotides of the opposite strand, with one designed to be one base short to incorporate the site-specific gap (oligonucleotide sequences listed below). 50 uM oligonucleotides were mixed together in 10 mM Tris-HCl, pH 7.5-8, 50 mM NaCl, 1 mM EDTA and then heated and cooled slowly to anneal. DNA was then ligated together using T4 DNA ligase. All oligonucleotides except for the one directly following the site of the desired missing base contained a 5’ phosphate group to enable ligation. Annealing and ligation was checked through non-denaturing and denaturing PAGE analysis on 5% acrylamide and 10% acrylamide, 8M urea gels respectively. DNA was then separated on an 8% acrylamide gel, extracted, and precipitated as described above.

*Oligonucleotides used to make DNA with gap near SHL-2 (-25):*

T1: 5’-Cy3-ctggagaatcccggtgccgaggccgctcaa-3’

T2: 5’-Phos-ttggtcgtagacagctctagcaccgcttaaacgcacgtacgcgctgtcc-3’

T3: 5’-Phos-cccgcgttttaaccgccaaggggattactccctagtctccaggcacgtg-3’

T4: 5’-Phos-tcagatatatacatcctgtgcatgtattgaacagcgaccttg-3’

T5: 5’-Phos-ccggtgccagtcggatagtgttccgagctcccactctagaggatccccgggtaccga-3’

B1: 5’-tcggtacccggggatcctctagagtg-3’

B2: 5’-Phos-ggagctcggaacactatccgactggcaccggcaaggtcgctgttcaataca-3’

B3 G-2: 5’-Phos-tgcacaggatgtatatatctgacacgtgcctggagactagggagtaatcccc-3’

B4 G-2: 5’-tggcggttaaaacgcgggggacagcgcgtacgtgcgtttaag-3’

B5: 5’-Phos-cggtgctagagctgtctacgaccaattgagcggcctcggcaccgggattctccag-3’

*Oligonucleotides used to make DNA with gap near SHL-6 (-56):*

T1: 5’-Cy3-ctggagaatcccggtgccgaggccgctcaa-3’

T2’: 5’-Phos-ttggtcgtagacagctctagcaccgcttaaacgcacgtacgcgct-3’

T3’: 5’-Phos-gtcccccgcgttttaaccgccaaggggattactccct-3’

T4’: 5’-Phos-agtctccaggcacgtgtcagatatatacatcctgtgcatgtattgaacagcgaccttg-3’

T5: 5’-Phos-ccggtgccagtcggatagtgttccgagctcccactctagaggatccccgggtaccga-3’

B1’: 5’-tcggtacccggggatcctctagagtgggagctcggaac-3’

B2 G-6: 5’-Phos-actatccgactggcaccggcaaggtcgctgttcaatacatgcacaggatgtatatatctg-3’

B3 G-6: 5’-cacgtgcctggagactagggagtaatccccttgg-3’

B4: 5’-Phos-cggttaaaacgcgggggacagcgcgtacgtgcgtttaag-3’

B5: 5’-Phos-cggtgctagagctgtctacgaccaattgagcggcctcggcaccgggattctccag-3’

Refolding of histone octamers was performed as described previously (*48-50*). Octamer containing H2A N38C was refolded in the presence of DTT to prevent disulfide formation. Oxidation and reduction were performed using CuPhe as described previously (*48*). Briefly, octamers were treated with freshly made 25 𝜇M Cu(II)SO_4_ and 100 𝜇M o-phenanthroline, followed by quenching with EDTA. The same octamer was reduced by dialyzing into buffer containing 100 mM DTT. Crosslinking efficiency was determined by quenching aliquots of the oxidized and reduced reactions with 50 mM iodoacetamide and running them on a non-reducing SDS-PAGE gel. Quantification of crosslinking was done by taking the ratio of the intensity of the crosslinked H2A band, which runs at a higher molecular weight, and the total of the crosslinked H2A band and the H2A/H2B band (fig. S12H). Because uncrosslinked H2A and H2B run at the same size, we assume that 100% crosslinking would result in a ratio of 0.5.

Nucleosomes and hexasomes were assembled using salt gradient dialysis (*23, 45*). Oxidized nucleosomes were assembled using buffers without reducing agent while reduced nucleosomes were assembled in buffers containing 3 mM TCEP. Nucleosomes were purified by ultracentrifugation with a 10-30% glycerol gradient. Hexasomes were purified using a Mini PrepCell (BioRad) with a 7% acrylamide gel based on the previous published method. Briefly, asymmetry of the 601 DNA sequence yields weaker affinity of one side of the DNA sequence for the H2A/H2B dimer versus the other side. Placing the flanking DNA adjacent to the weaker side allows the assembly of hexasomes with the H2A/H2B dimer missing proximal to the longer flanking DNA. The hexasomes are further purified away from nucleosomes through a prep-cell as described by Levendosky and Bowman (*23, 24*).

Native gel-based remodeling assay

All remodeling reactions were done under single turnover conditions (enzyme in excess of nucleosomes) with saturating INO80 and ATP. The reactions were carried out at 30 °C. Briefly, 60 nM INO80 WT was incubated with 15 nM nucleosomes or hexasomes in reaction buffer (40 mM Tris-HCl, pH7.5, 50 mM KCl, 1.1mM free MgCl_2_, 0.02% NP-40, and 7% glycerol) for 10 minutes at 30 °C before adding 1mM ATP· MgCl_2_ to start the reaction. The no ATP control was taken at the last time point of the reaction. The reaction samples taken at specific time points were quenched with excess plasmid DNA and ADP. Samples were resolved on a native PAGE gel (6% acrylamide, 0.5X TBE) ran for 3 hours at 125V. Gels were scanned on a Typhoon Imager (GE Life Sciences) and quantified by densitometry using ImageJ. The fraction of nucleosome products was determined by the ratio of slower-migrating nucleosomes (everything above unremodeled nucleosomes) to the total nucleosome intensity. Using Prism 7 (GraphPad), data were fit to a single-phase exponential decay model (Equation 1),

$y=\left( y_{0}-p \right)e^{-k_{obs}t}+p$,

where *y_0_* is the initial fraction product, *k_obs_* is the observed rate constant, and *p* is the fraction product at the plateau. Due to the much slower rates of reactions containing +80N Gap @ SHL-2, +80N Gap @ SHL-6, and +80H Gap @ SHL-2, the plateau, *p*, was not empirically reached in the timeframe used. Therefore, to fit these data, we used a single-phase exponential decay that constrained *p* to a constant equal to the average fraction remodeled at the plateau of +80N or +80H reactions from fitting with no constraints. The assumption behind our approach is that when these very slow constructs are fully remodeled, the proportion remodeled will resemble that of our +80N and +80H constructs.

Amine functionalized GO grids preparation

Preparations and functionalization of Graphene Oxide (GO) grids were performed following the previously described protocol (*51, 52*). Briefly, in a glass petri dish (60 mm in diameter, 15 mm in height) an epoxy coated stainless steel mesh stand was placed at the bottom and DI water was filled to the top. 300 Mesh, R1.2/1.3 Au Quantifoil grids were placed on the mesh stand with carbon side facing upward. Using a syringe, the GO solution (230 ul in total volume) was spread onto the water surface. After draining the water, the GO coated grids were dried at room temperature for use. GO covered grids were then submerged in 10 mM ethylenediamine (Sigma-Aldrich E26266) solution diluted in dimethyl sulfoxide (DMSO) and incubated for 5 h at room temperature. The grids were washed thoroughly twice with DMSO without ethylenediamine, twice with autoclaved water, twice with ethanol, and dried under ambient conditions. Amino modified grids were stored dry at −20 °C until use.

Electron microscopy sample preparation and data collection

Freshly prepared INO80 was mixed with either hexasome or nucleosome in 1:1 molar ratio, and then incubated at 30 °C for 30 min, after which the buffer was exchanged to EM buffer (25mM HEPES, pH 7.5, 100mM KCl, 2mM MgCl_2_, 2mM DTT, 1% glycerol). The final concentration of the complex was ~0.05- 0.1 µM and no nucleotide was added. All cryo-EM grids were prepared using functionalized GO-amine grids.

Negative staining of the complex was performed with 0.75% uranium formate, following an established protocol (*53*). Grids were examined using an FEI T12 microscope operated at 120 kV, and images were recorded using a 4k x 4k charge-coupled device (CCD) camera (UltraScan 4000, Gatan).

To prepare cryo-grids, samples (3μl at 0.2 μM) were loaded onto the amine modified GO grids, and then blotted for 4 s before being plunge-frozen in liquid ethane cooled by liquid nitrogen using a FEI Vitrobot IV with the sample chamber set at 8°C and 100% humidity. The blotting force was 0, using ø 55/20 mm blotting filter paper from TED PELLA. Grids were examined and screened using an FEI Tecnai Arctica operated at 200 kV and equipped with a Gatan K3 camera. All cryo-EM datasets were collected on a Titan Krios at the UCSF Cryo-EM Center for Structural Biology operated at an acceleration voltage of 300 kV and equipped with a BioQuantum energy filter (slit width set to 20 eV) and a K3 direct electron detector (Gatan).

All cryo-EM datasets were collected using SerialEM (*54*). Multishot collection (3×3 arrays) was performed with using beam-tilt compensation and the maximum image shift is 3.5 microns. All images were acquired with a nominal magnification of 105 K, resulting in a pixel size of 0.4175 Å. Defocus range was set from -1.0 μm to -2.0 μm. For the INO80-hexasome sample, 18,991 images were collected, each was dose-fractionated to 117 movie frames with a total exposure time of 5.9 s, resulting in a total fluence of ~67 electrons per Å^2^. For the INO80-nucleosome sample, 8,653 images were collected, each was dose-fractionated into 80 movie frames with a total exposure time of 2.024 s, resulting in a total fluence of ~43 electrons per Å^2^.

Image processing

For the dataset of the INO80-hexasome sample, a total of 18,991 movie stacks were motion corrected and dose weighted using MotionCor2 (*55*). The CTF parameters were estimated and all subsequent 2D and 3D classification were performed using cryoSPARC (*56*). 500 micrographs were randomly selected to generate a template. In brief, 624,536 particles were picked by cryoSPARC blob picker and were subject to ab-initio reconstruction and multi-round heterogenous refinement. From one good class showing clear features of INO80 bound to the hexasome, we generated 16 different projection images for template picking, yielding 7,831,514 particles from all micrographs. After multiple rounds of heterogenous refinement, a final 1,220,910 particles were selected to calculate a reconstruction of the INO80-hexasome complex with a global resolution of 2.8 Å. This reconstruction showed clear structural features of INO80, but the hexasome was not well resolved.

This particle stack was then exported to RELION (*57*). 3D refinement (initial low-pass filter: 10 Å; mask diameter: 360 Å; reference mask: no) was applied to get the new reconstruction, after which segment map function in Chimera (*58*) was used to split this reconstruction and generate a map only containing the Arp5-Ies6 module and hexasome. A mask containing the Arp5-Ies6 module and hexasome was then generated from this map (initial threshold: 0.0001; extend_inimask: 4; width_soft_edge: 6). Particle subtraction function in RELION was applied to create the particle stacks that only contain the Arp5-Ies6 module and hexasome in theory (do center subtracted images on mask: yes; new box size: 240), followed by a relion reconstruction to obtain a reference map (maxres: 10; ctf: yes). A mask was then generated for the following procedure (initial threshold: 0.001; extend_inimask: 4; width_soft_edge: 6). 3D classification of background subtracted particles (reference mask: generated last step; initial low-pass filter: 10 Å; mask diameter: 200 Å; regularisation parameter T: 3; number of iterations: 50; number of classes: 5; angular sampling interval: 1.8º; perform local angular searches: yes; local search range: 18) producing a new map of 560,912 particles with clear Arp5-Ies6 and hexasome features. Then, a 3D reconstruction of the entire INO80-hexasome complex (initial low-pass filter: 10 Å; mask diameter: 360 Å; reference mask: no) was calculated from this same subset of particles and further refined, in which the densities of both INO80 and hexasome are improved compared with the previous round. We then performed another round of background subtraction with a mask on the hexasome (initial threshold: 0.001; extend_inimask: 4; width_soft_edge: 6), followed by classification with local alignment focused entirely on the hexasome (reference mask: generated last step; initial low-pass filter: 10 Å; mask diameter: 200 Å; regularisation parameter T: 3; number of iteration: 50; number of classes: 5; angular sampling interval: 1.8º; perform local angular searches: yes; local search range: 18) . Three major classes were identified, among which the main difference is the hexasome orientation, mainly reflecting different binding positions of INO80 on the hexasome.

For each class, we then further refined the whole complex and hexasome to higher resolutions by using either cryoSPARC or cisTEM (*59*). In cryoSPARC, non-uniform refinement with default setting was performed. In cisTEM, generate 3D function with default setting was applied. The final 3D maps were sharpened by DeepEMhancer (*60*), except for the hexasome of class 1. Lastly, reconstructions of INO80 and the hexasome were assembled into a composite map for model building and figure generating. For both class 1 and class 3, we also generated a mask around the flanking DNA for further focused 3D classification, producing a clear density that allows docking of the crystal structure of Arp8-N-actin-Arp4 into the density map (fig. S2B).

For the dataset of the INO80-nucleosome sample, a total of 8,653 movie stacks were motion corrected and dose weighted using MotionCor2. The CTF parameters were estimated and all subsequent 2D and 3D classification were performed using cryoSPARC. 3,625,796 particles were picked by cryoSPARC template picker. The remaining parts of image processing were performed following the procedure described above.

In all reconstructions, no CTF refinement was performed. For each reconstruction, the numeric resolution was determined from Fourier Shell Correction (FSC) using criterion of FSC = 0.143 (*61*). In addition, directional FSC (dFSC) (*62*) was used to evaluate the directional uniformity of all reconstructions, as reported in Figure S3 and S8.

Model building

For the model building, the initial model was generated by fitting the available coordinates into our cryo-EM density maps by using Chimera (*58*). These coordinates include the INO80 core (with its sequence changed to that of *S. cerevisiae* by Alphafold (*63*) and ccp4em), the crystal structure of Arp8-N-actin-Arp4 and the model of a hexasome (PDB: 6FML, 8A5O, 6ZHY (*14, 36, 40*)). The inconsistent parts were then manually built and refined in coot (*64*). The structures were refined using Phenix (*65*) with secondary structure constraints. Model building of INO80-nucleosome complexes was performed following the same procedure, except that the atomic model of a nucleosome (PDB: 1KX5) (*66*) was used.

Summary of parameters used in data collection and model building are in Table S1 (INO80-hexasome) and Table S2 (INO80-nucleosome).

Supplementary Text

Nomenclature definition of +80H hexasome and +80N nucleosome

+80H Hexasome and +80N nucleosome are assembled on the same DNA templates containing the 147 bp 601 nucleosome positioning sequence with 80 bp of additional DNA. Hexasomes are asymmetric and lack the entry-site proximal H2A-H2B. We define the entry and exit DNA sites as the sites closest and farthest from the flanking DNA, respectively. Further, given the additional DNA that is unwrapped in a hexasome, we define flanking DNA as the additional DNA beyond the 147 bp of the 601 sequence, and free DNA as the DNA that is not bound to histones. Thus, +80N has 80 bp of free DNA while +80H has ~ 115 bp of free DNA because of the additional ~35 bp of DNA that is unwrapped from removal of an H2A-H2B dimer (Fig. 1A, and fig. S1, A and B).

Cryo-EM structures of the INO80-hexasome complex

We determined cryo-EM structures of INO80-hexasome by single particle cryo-EM and captured three major conformational snapshots of the complex. The major conformation (class 3), which contains the largest portion of particles in our dataset, has a global resolution of 2.6Å and a local resolution for the hexasome at 2.9Å, allowing accurate model building for most of the complex (Fig. 1, B and C, and figs. S2 to S5). Two other conformational snapshots were isolated from the same dataset by focused classification centered on the hexasome (figs. S2 and S6, A and B). The overall resolutions of these two reconstructions are 3.0Å (class 1) and 2.8Å (class 2), with the local resolution of the hexasome at 6.7Å and 3.2Å, respectively, sufficient to accurately position the atomic model of a hexasome (fig. S3, A to D).

As described in the main text, the whole INO80 complex is grouped into four modules: Rvb module, Arp8 module, Ino80 module and Arp5 module. Common features that are resolved in all three conformational snapshots are described in the following: The Rvb module is well-resolved in all three classes with an almost identical conformation. It contains the heterohexamer formed by the Rvb1 and Rvb2 that serves as a scaffold to assemble other subunits (Fig. 1B, and fig. S4A). Although no nucleotide was added during sample preparation, clear density for ADP is seen in all nucleotide binding pockets of Rvb1/Rvb2 (fig. S4C). The two RecA lobes in the ATPase domain (N-lobe and C-lobe) are interrupted by a large insertion, which can clearly be seen threaded through the Rvb1/Rvb2 hexamer similar to previous INO80-nucleosome structures (*12, 14*). The Arp8 module which is known to interact with Ino80 through its HSA domain is resolved at a lower resolution and insufficient for model building but sufficient for docking the atomic model (PDB: 8A5O). Note that the Taf14 subunit of Arp8 module is not part of the atomic model, nor it is seen in our cryo-EM density map, even at a lower density counter level, suggesting that may be intrinsically flexible. For the Ino80 module, Ino80^NTD^ is not visible, Ino80^HSA^ is only partially resolved. For the Ino80^ATPase^, the C-lobe of is clearly resolved but its N-lobe is more flexible, resolved only sufficiently for docking the atomic model (PDB: 6FML, (*14*)). The flexibility of the N-lobe of the Ino80^ATPase^ may be a consequence of nucleotide not being added to our sample. The core of the Arp5 subunit is well resolved with its DNA binding domain (Arp5^DBD^) interacting with hexasomal DNA at SHL+2 (fig. S5, C and D) and with density for an ATP molecule in the nucleotide binding pocket (fig. S4D). Furthermore, similar to the previously published INO80-nucleosome structure (*12, 14*), the C-terminal HIT-like domain of Ies6 forms a stable contact with Rvb1/2, while the rest of Ies6 wraps around Arp5 (fig. S5E). Nhp10 module is another module that is also present in the complex but is not resolved, suggesting their flexibility.

One available atomic model of a hexasome was determined with a short peptide bound (PDB: 6ZHY, (*36*)), which we approximate as representing an unbound hexasome. The hexasome bound by INO80 is well defined in all three structures (figs. S3, B, D and F, and S4B), retaining the canonical structural features of a hexasome seen in previous structures (*36, 67, 68*).

Cryo-EM structures of the INO80-nucleosome complex

The prior INO80-nucleosome structures were determined with human and *C. thermophilum* INO80, while we use *S. cerevisiae* INO80*.* To control for any species related differences, we also determined structures of *S. cerevisiae* INO80 bound to a nucleosome without added nucleotide. 3D classification reveals two major conformations, with overall resolutions of 3.5Å for class 1 and 3.4Å for class 2, and the local resolutions for the nucleosomal region of 3.5Å and 3.3Å, respectively (figs. S7, S8, and S9, A and C). The INO80 structure is almost identical between these two states, with an rmsd of 0.4Å. The overall architecture of *S. cerevisiae* INO80 on a nucleosome is also similar to that in previous INO80-nucleosome structures (*12, 14*). The C-terminal tail of Ies6 is better resolved in class 2, and makes a close interaction with H2B, correlating with a shift of H2B towards Ies6 by ~1Å, as measured from the position of Y118 in H2B (fig. S9E). We see also much less DNA unwrapping for each INO80-nucleosome class.

Engagement of Arp8 module with flanking DNA

Prior studies have suggested that the Arp8 and Nhp10 modules bind flanking DNA (*69-71*). To understand the role of these modules in hexasome and nucleosome sliding we compared their locations in our structures. In both the INO80-hexasome and INO80-nucleosome structures, we can resolve the Arp8 module after focused classification but with lower resolution (figs. S2B, S5, A and B, S7B, and S9B). Nonetheless, the quality of the density maps allows us to place the available atomic model of the Arp8 module (PDB: 8A5O (*40*)) into the cryo-EM density maps and to build an atomic model together with the rest of INO80. However, we did not resolve density of Nhp10 in our cryo-EM density maps, likely because it binds to flanking DNA further beyond where Arp8 module binds and is more flexible. Furthermore, similar to a previous report, we did not resolve Taf14, a subunit in the Arp8 module, likely because of its conformational flexibility (*40*).

Differences in the interactions between Arp5 module with a hexasome and a nucleosome

Additionally, we observe that the Arp5/Ies6 module makes substantially different contacts in the hexasome compared to a nucleosome. In prior INO80-nucleosome structures and the structures obtained here, the Arp5 module makes interactions with the acidic patch of the entry-site proximal H2A-H2B dimer using its grappler domain (fig. S10, A and D) and with nucleosomal DNA between SHL-2 and -3 using its DNA binding domain (fig. S10B) (*12, 14*). In Class 1 of the INO80-nucleosome complex, the grappler is not as well resolved as in Class 2, suggesting that the grappler may be positioned too far away to fully engage the acidic patch. Ies6 wraps around Arp5 and interacts with the H2A-H2B dimer and DNA at SHL-2 (fig. S10C). In contrast, on a hexasome, the Arp5^DBD^ binds DNA between SHL+1 and +3 (fig. S5, C and D) and the Arp5^grappler^ appears to interact with the exposed H3-H4 tetramer and flanking DNA at the entry site (fig. S5F). In addition, on a nucleosome, we observe an Arp5 interaction with flanking DNA as seen previously ((*40*); fig. S10E). In comparison, on a hexasome, Arp5 interacts with DNA unwrapped from the histone core (fig. S5G). Ies6 maintains similar interactions with Arp5, but no longer interacts with the H2A-H2B dimer as it is missing in a hexasome. Its interaction with DNA changes to the SHL+1/+2 location. Thus, unlike in a nucleosome, where the Arp5 grappler contacts the H2A-H2B acidic patch, in a hexasome, the grappler contacts the exposed H3-H4 surface.

**
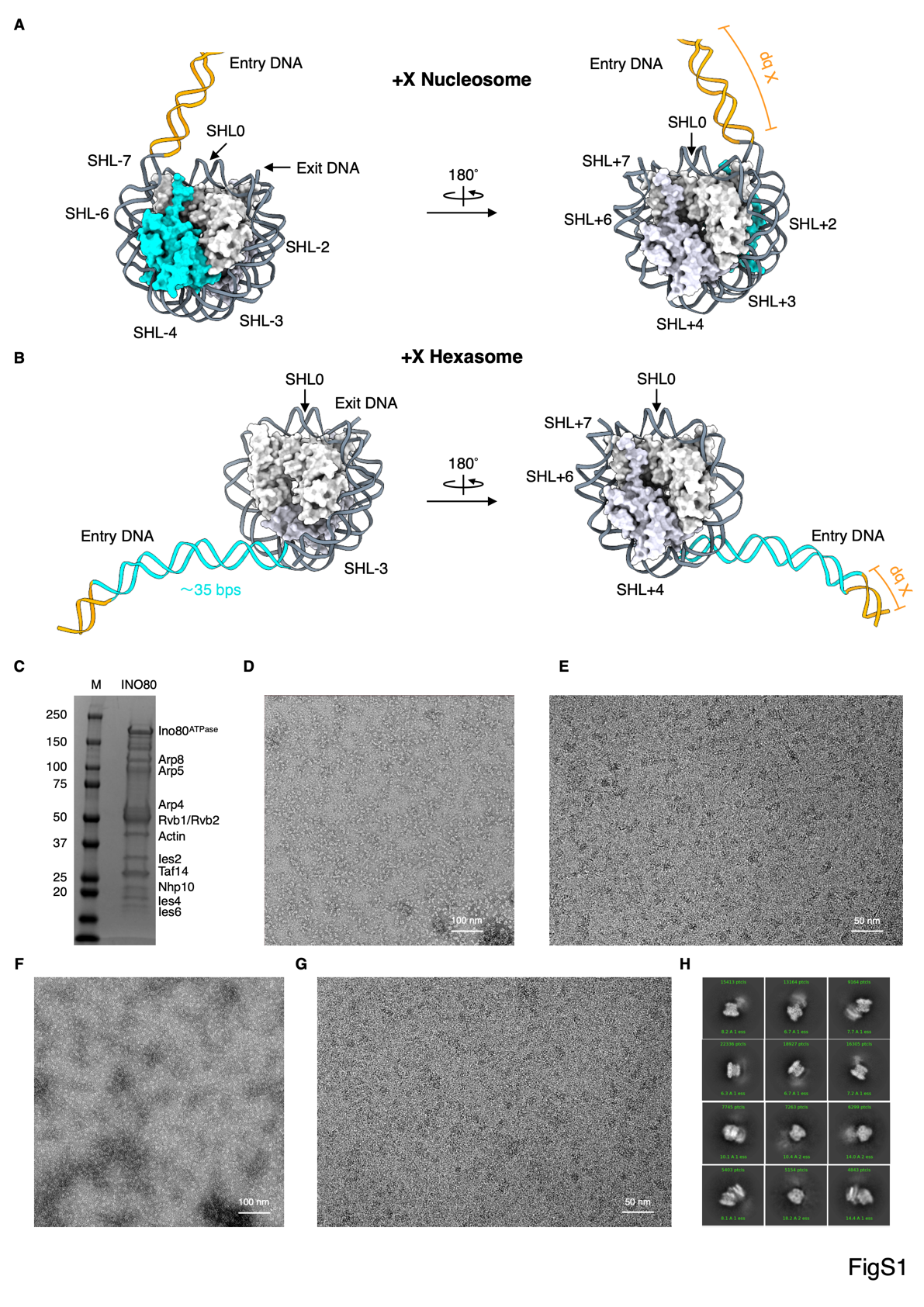
**

**Fig. S1. Preparation of INO80-nucleosome and INO80-hexasome samples.**

(A-B) Models of +X Nucleosome (A) and +X Hexasome (B).

(C) SDS-PAGE gel of purified *S. cerevisiae* INO80 complex.

(D) Negative staining of the INO80-hexasome sample.

(E) Representative cryo-EM micrograph of the INO80-hexasome complex.

(F) Negative staining of the INO80-nucleosome complex.

(G) Representative cryo-EM micrograph of the INO80-nucleosome complex.

(H) Representative 2D average image of the INO80-nucleosome complex.


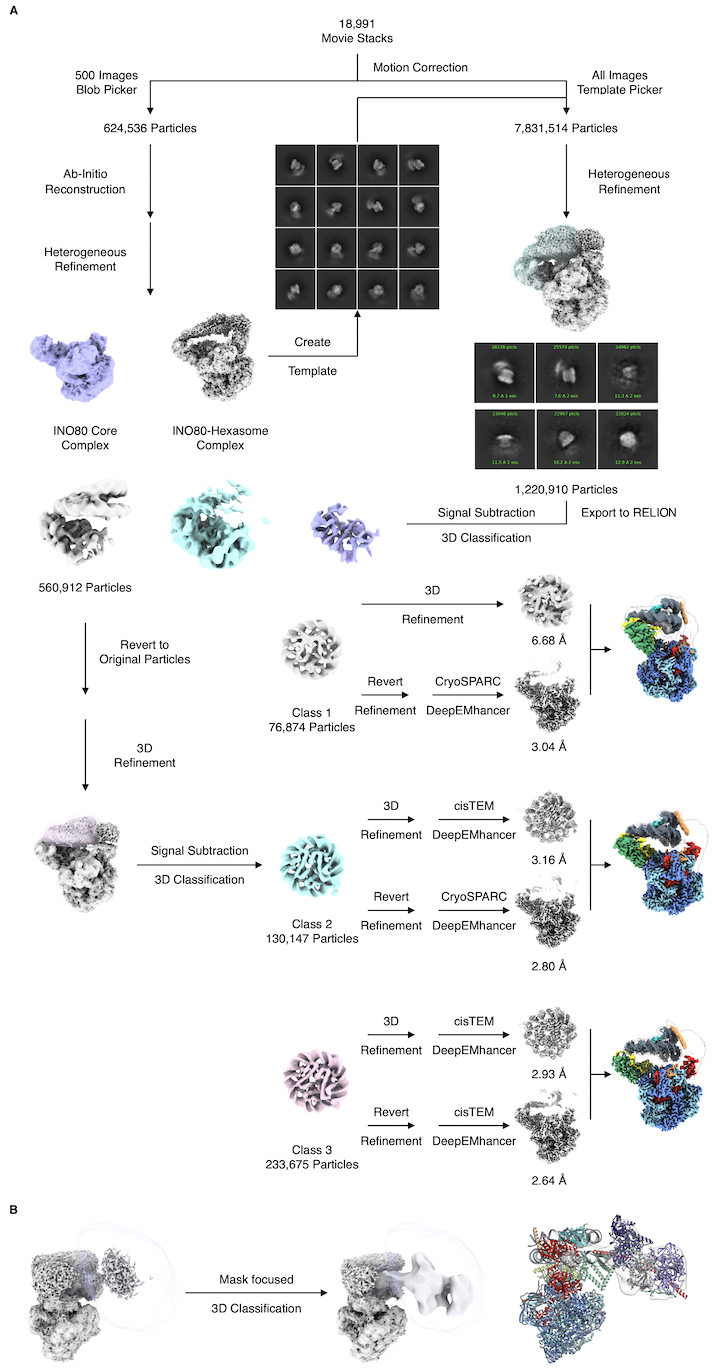


**Fig. S2. Image processing of the INO80-hexasome dataset**.

(A) A flow-chart outlining the processing of the INO80-hexasome cryo-EM dataset.

(B) Focused classification near flanking DNA produced density of the Arp8 module.

**
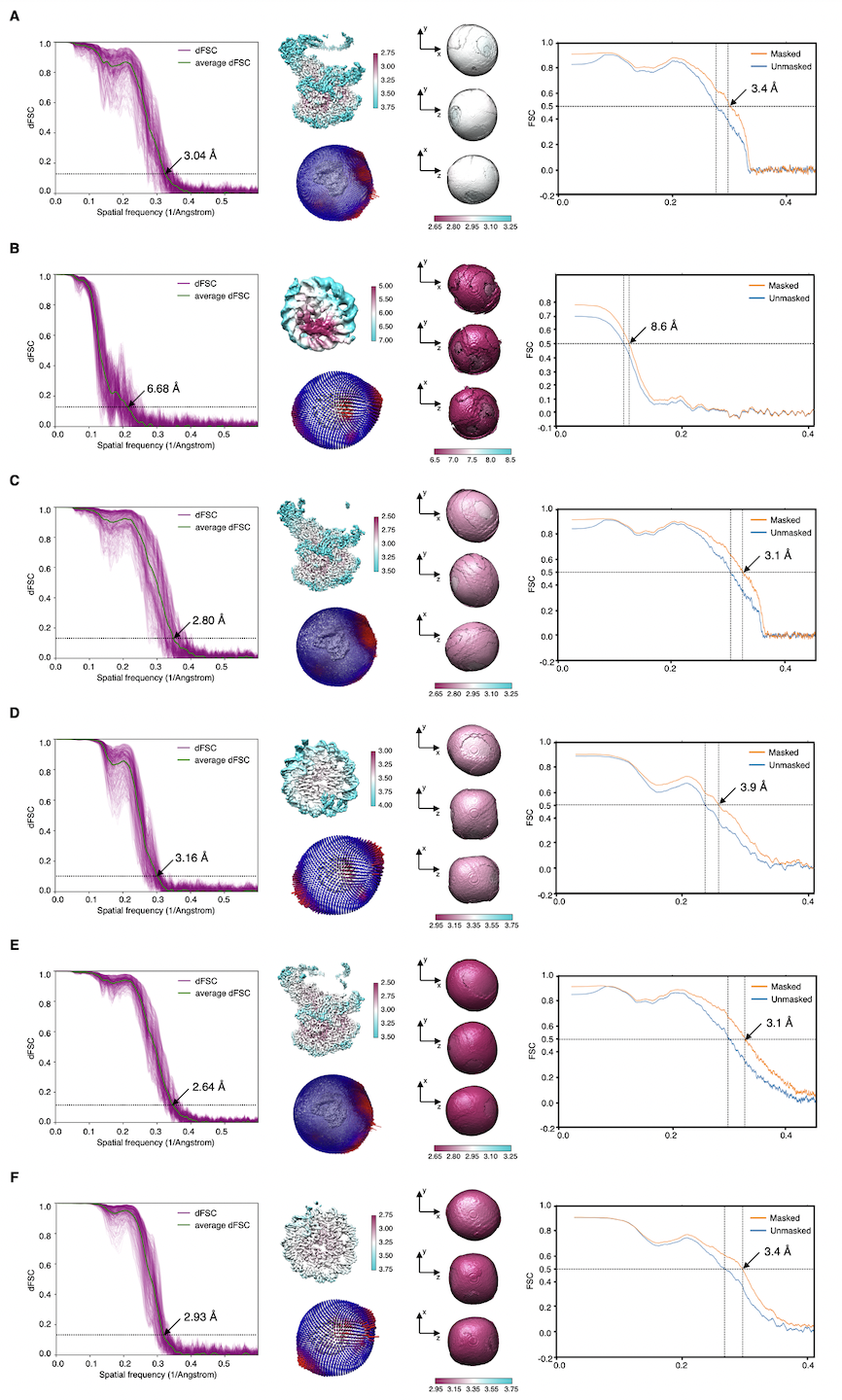
**

**Fig. S3. Resolution estimation of the INO80-hexasome structures.**

All panels contain, from left to right, directional Fourier shell correlation (dFSC) curves of final maps with resolution determined by the FSC criterion of 0.143, cryo-EM map colored by local resolution with resolution scale bar, angular distributions of particles, three-dimensional representations of the dFSC, and model-map FSC plots calculated by Phenix between the map and the model.

(A-B) Class 1 of INO80-hexasome complex (A) and the corresponding hexasome (B).

(C-D) Class 2 of INO80-hexasome complex (C) and the corresponding hexasome (D).

(E-F) Class 3 of INO80-hexasome complex (E) and the corresponding hexasome (F).

**
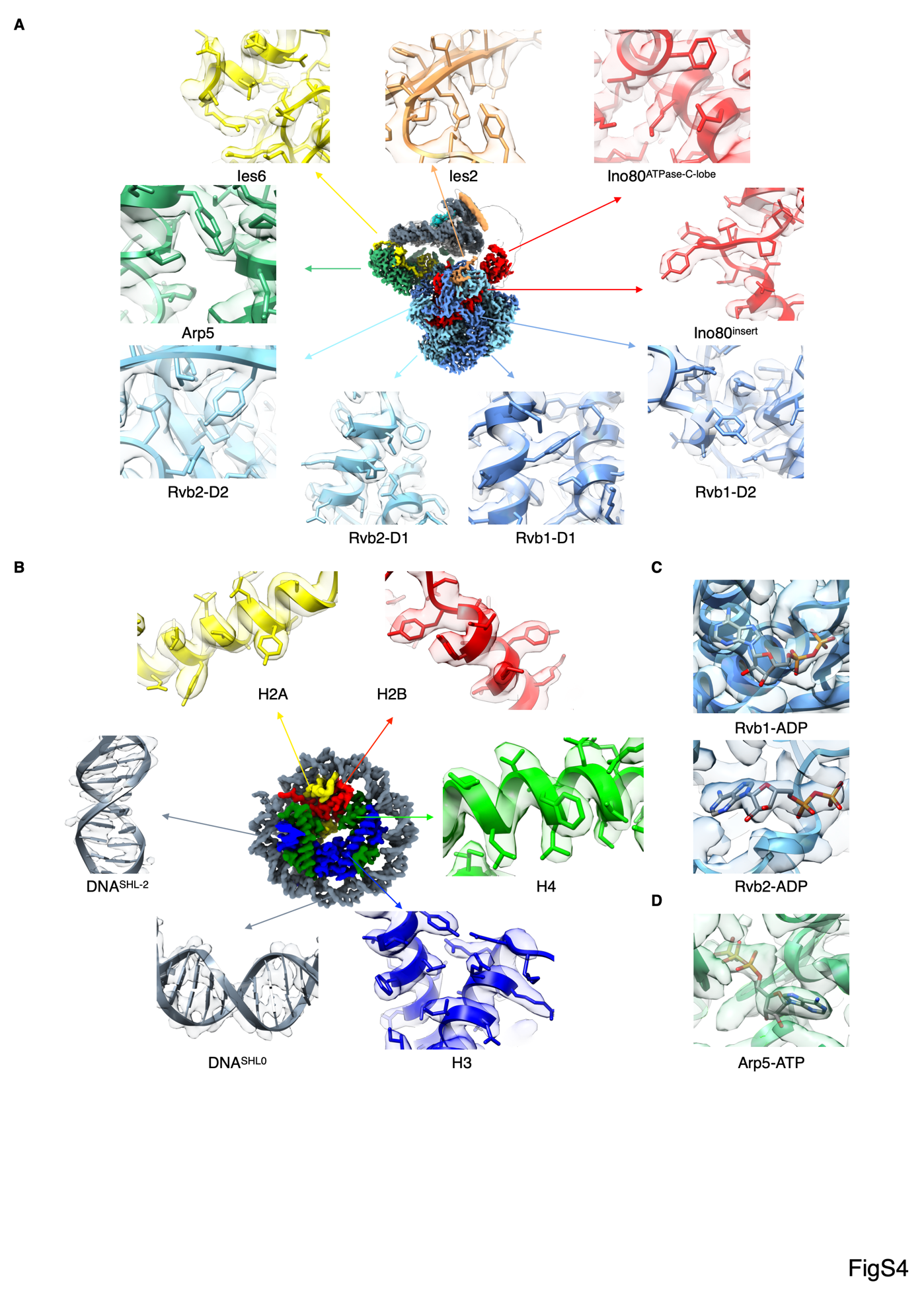
**

**Fig. S4. Representative densities of the INO80-hexasome complex**.

(A) Representative densities of INO80 from class 3 of the INO80-hexasome complex.

(B) Representative densities of the hexasome region from class 3 of the INO80-hexasome complex.

(C) Nucleotide densities seen in all six nucleotide binding pockets of Rvb1/Rvb2 heterohexamer are modeled as ADP.

(D) Nucleotide density seen in the Arp5 nucleotide binding pocket is modeled as ATP.


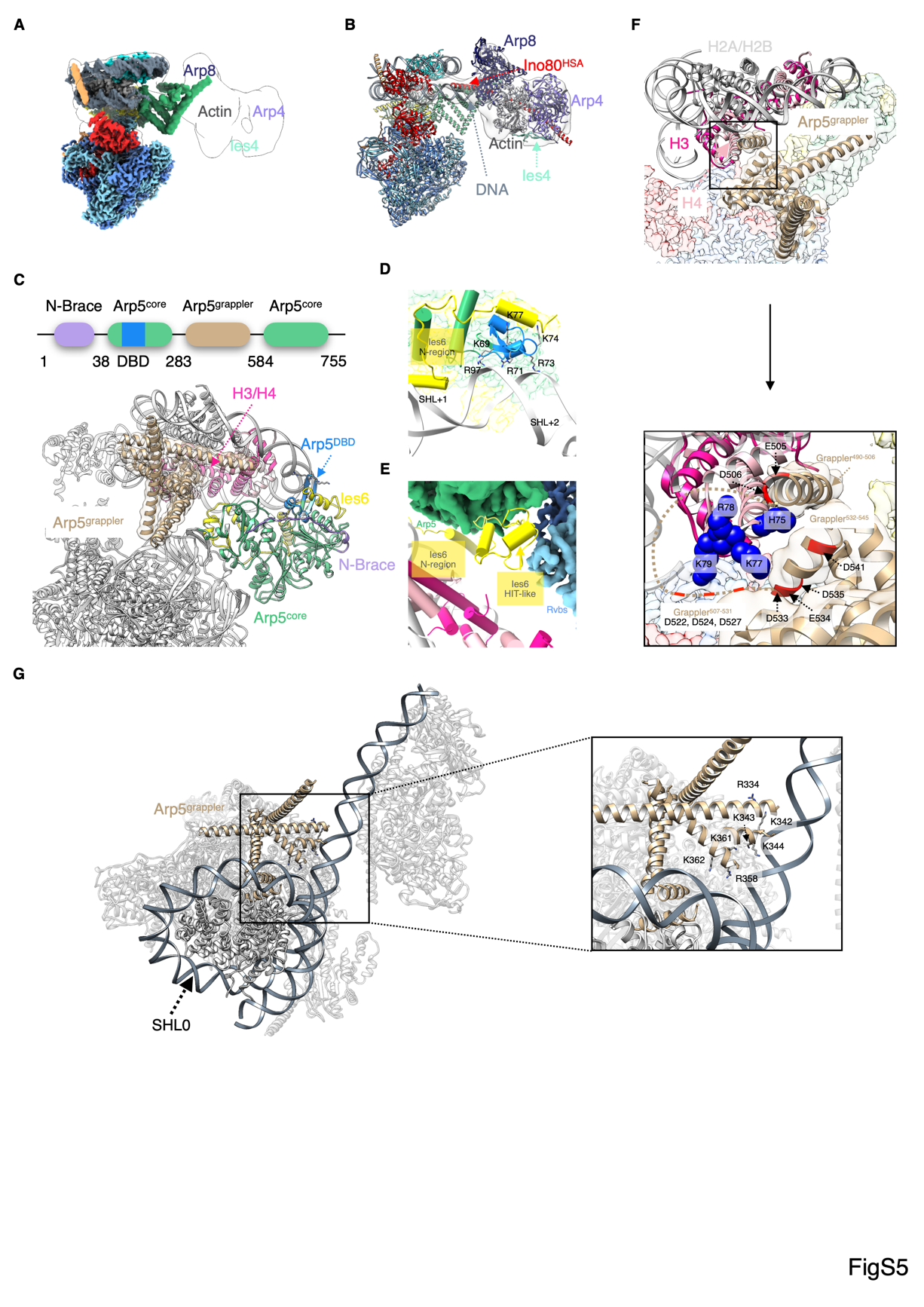


**Fig. S5. Arp5 and Arp8 modules in the INO80-hexasome structure.**

(A) Cryo-EM map of the INO80-hexasome complex (class 3) with density of the Arp8 module shown as transparent.

(B) Atomic model from the INO80-hexasome complex (class 3) shown in the same view as (A), with a model of the Arp8 module (PDB: 8A5O) docked into the density map.

(C) Domain arrangement (upper) and atomic model (bottom) of the Arp5 module.

(D) An enlarged view showing interactions between Arp5^DBD^ and DNA.

(E) An enlarged view showing interactions between Ies6, Arp5 and Rvb1/Rvb2.

(F) Two enlarged views of the Arp5^grappler^ and H3-H4 tetramer highlighting residues that may form contacts. Acidic residues: red; basic residues: blue, sphere.

(G) Two enlarged views of the Arp5^grappler^ and DNA unwrapped from the histone core highlighting Arp5 residues that may form contacts with the unwrapped DNA.

**
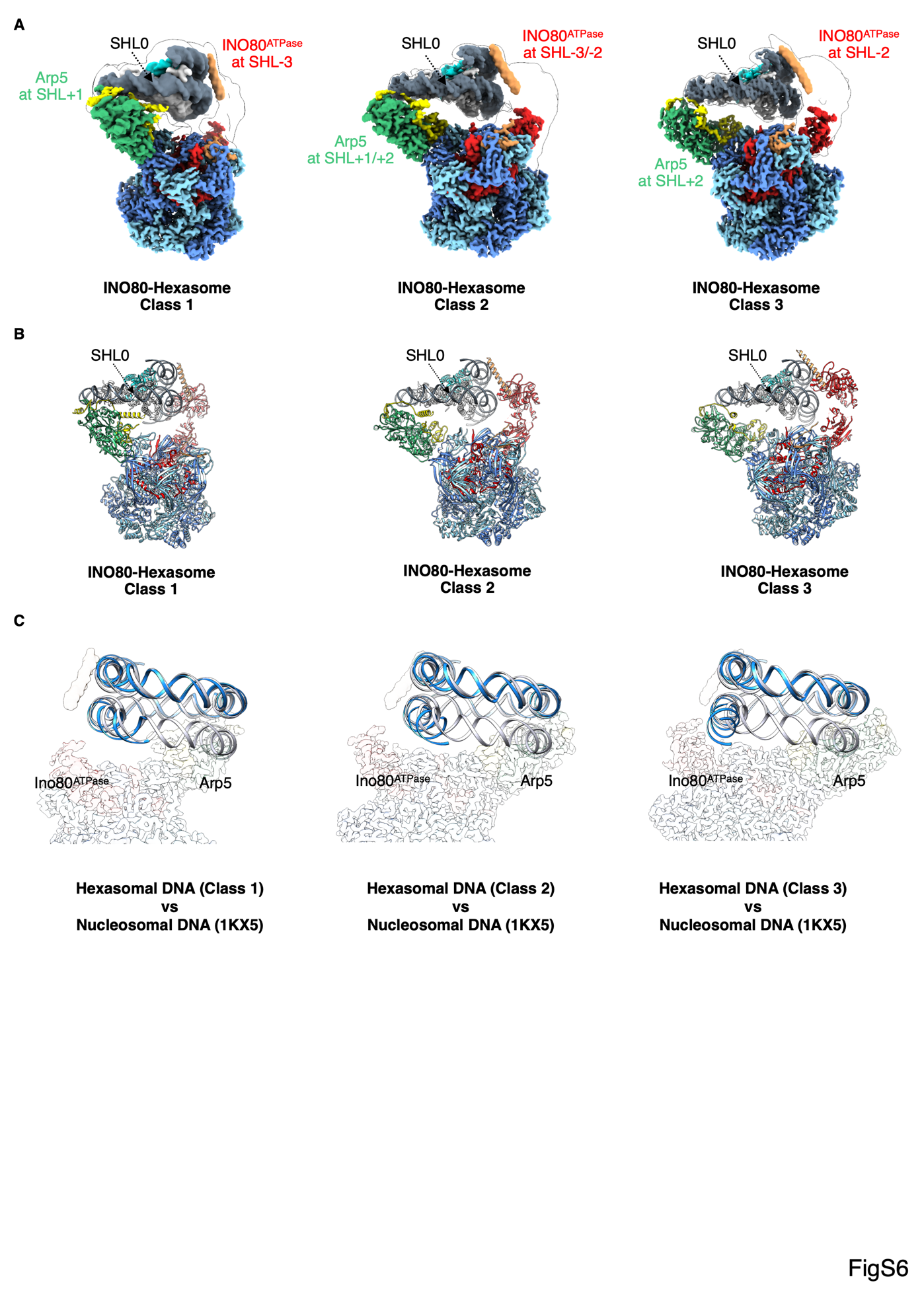
**

**Fig. S6. Comparison of three INO80-hexasome conformational snapshots.**

(A) Cryo-EM density maps of the INO80-hexasome complex in three different conformations. Maps are aligned by the hexasome orientation. Ino80^ATPase^ and Arp5 binding sites are labeled.

(B) Comparison of atomic models of the three classes of the INO80-hexasome complex, where the hexasome dyads are aligned using the H3-H4 tetramers.

(C) Comparison of the hexasomes from the three classes of the INO80-hexasome complex with the crystal structure of a free nucleosome aligned by the histone core. Hexasomal DNA: blue; Nucleosomal DNA: gray.


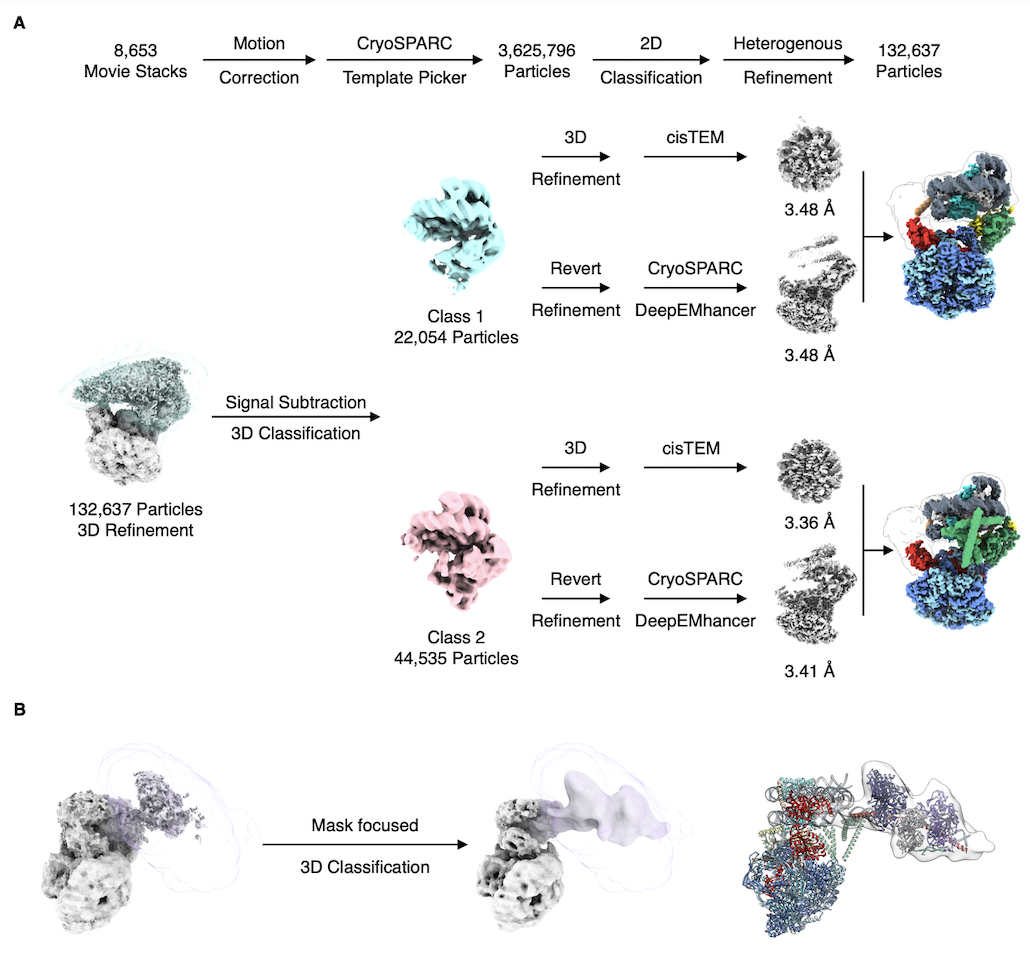


**Fig. S7. Image processing of the INO80-nucleosome**.

(A) A flow-chart outlining the processing of the INO80-nucleosome cryo-EM dataset.

(B) Focused classification near flanking DNA produced density of the Arp8 module.

**
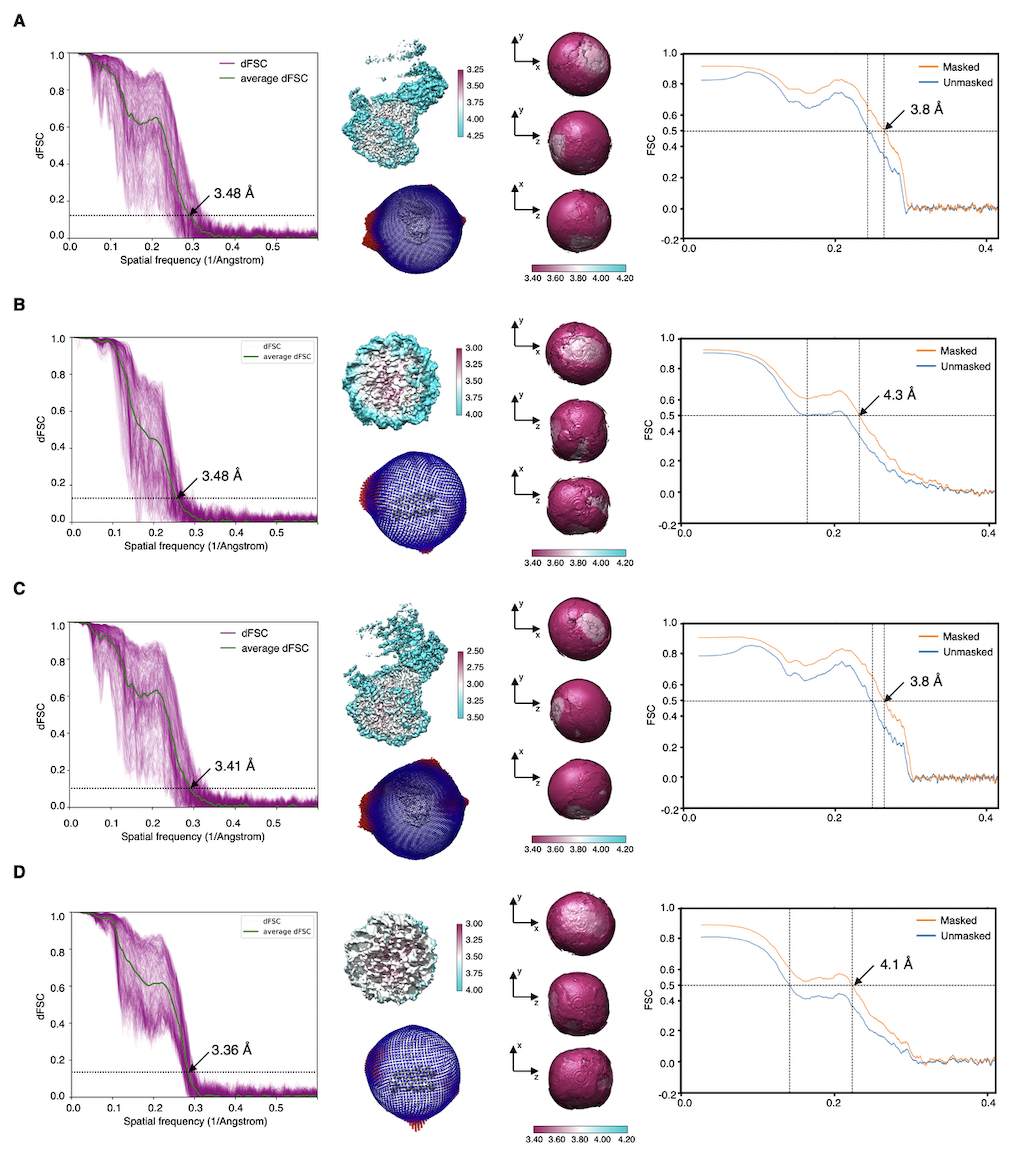
**

**Fig. S8. Resolution estimation of the INO80-nucleosome structures.**

All panels contain, from left to right, directional Fourier shell correlation (dFSC) curves of final maps with resolution determined by the FSC criterion of 0.143, cryo-EM map colored by local resolution with resolution scale bar, angular distributions of particles, three-dimensional representations of the dFSC, and model-map FSC plots calculated by Phenix between the map and the model.

(A-B) Class 1 of INO80-nucleosome complex (A) and the corresponding nucleosome (B).

(C-D) Class 2 of INO80-nucleosome complex (C) and the nucleosome (D).

**
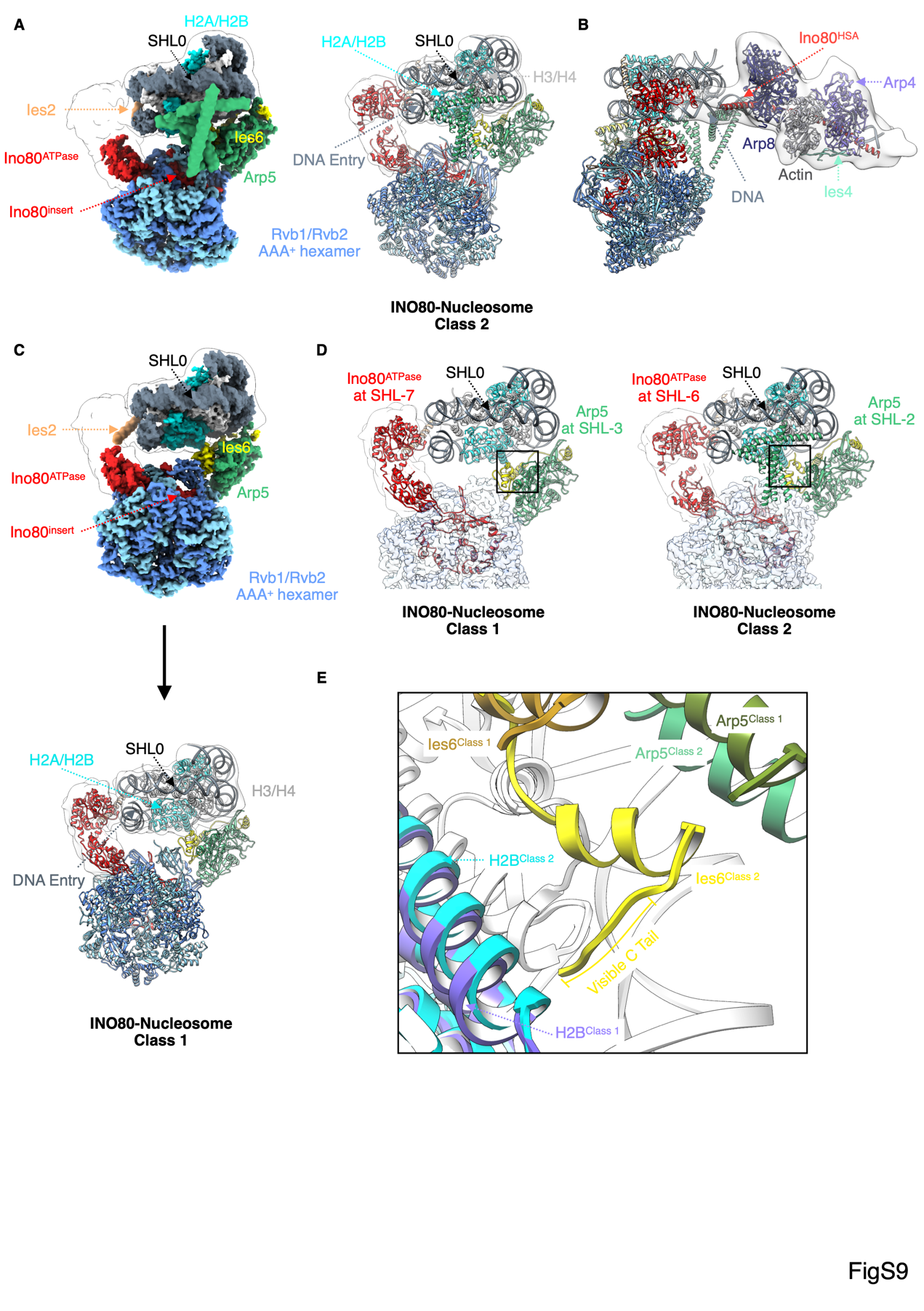
**

**Fig. S9. Structure of the INO80-nucleosome**.

(A) Cryo-EM map and atomic model of the *S. cerevisiae* INO80-nucleosome complex (class 2).

(B) Atomic model of the INO80-nucleosome complex (class 2) with a model of the Arp8 module (PDB: 8A5O) docked into the density map.

(C) Cryo-EM map and atomic model of the *S. cerevisiae* INO80-nucleosome complex (class 1).

(D) Two conformational snapshots of INO80-nucleosome complex aligned by the nucleosome. Binding location of Ino80^ATPase^ and Arp5 are labeled.

(E) Enlarged view of the boxed areas in (D) shows overlay of nucleosome atomic models in the two conformations. In class 2, H2B near flanking DNA is shifted slightly and the C terminal tail of Ies6 becomes visible.


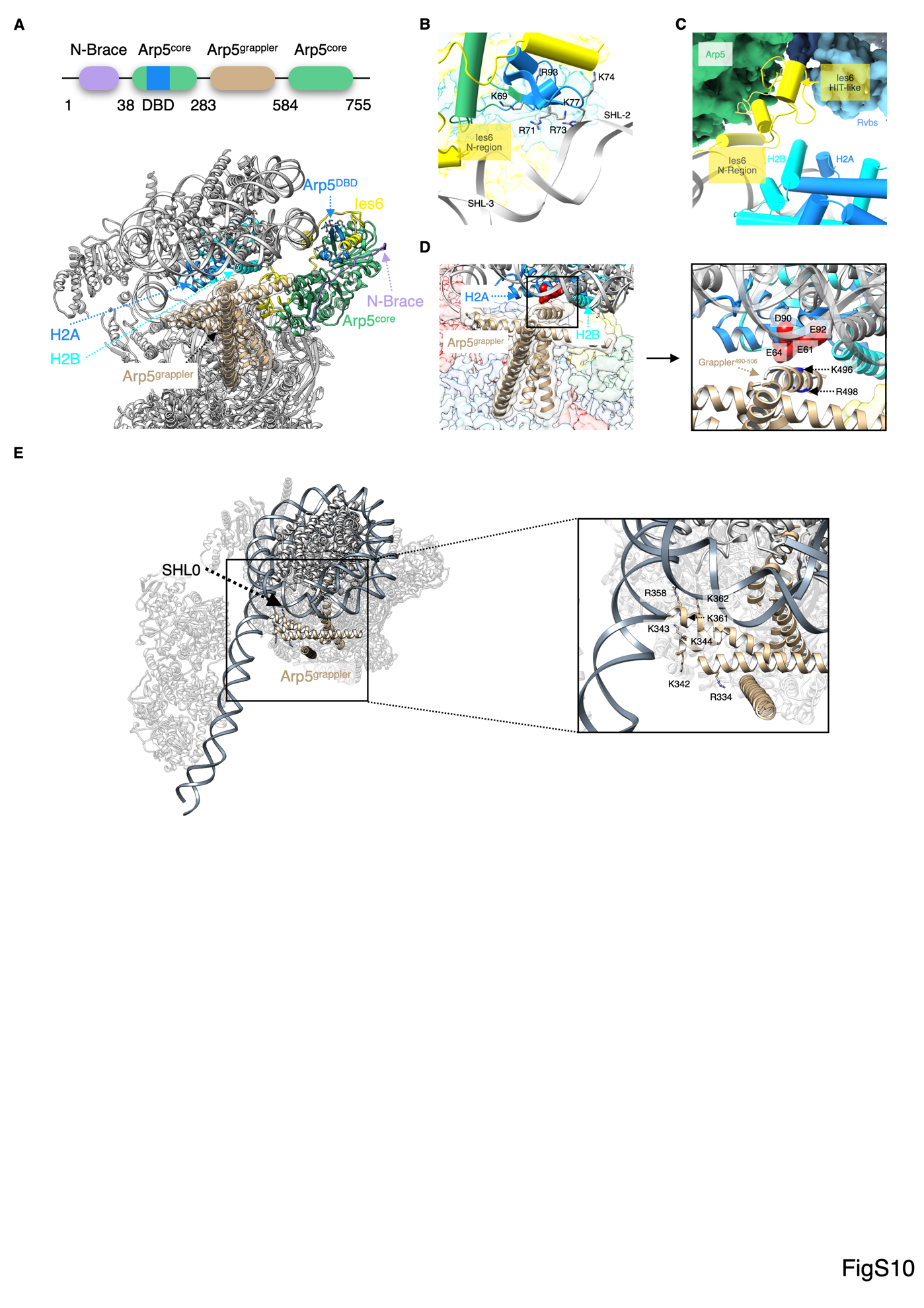


**Fig. S10. Arp5 interactions in the INO80-nucleosome structure**.

(A) Domain arrangement (upper) and atomic model (bottom) of the Arp5 module.

(B) An enlarged view showing interactions between Arp5^DBD^ and DNA.

(C) An enlarged view showing interactions between Ies6, Arp5 and Rvb1/Rvb2.

(D) Two enlarged views of the Arp5^grappler^ and the H2A-H2B dimer proximal to the flanking DNA highlighting residues that may form contacts. Acidic residues: red, sphere; basic residues: blue.

(E) Two enlarged views of the Arp5^grappler^ and flanking DNA of the nucleosome highlighting Arp5 residues that may form contacts with the flanking DNA.

**
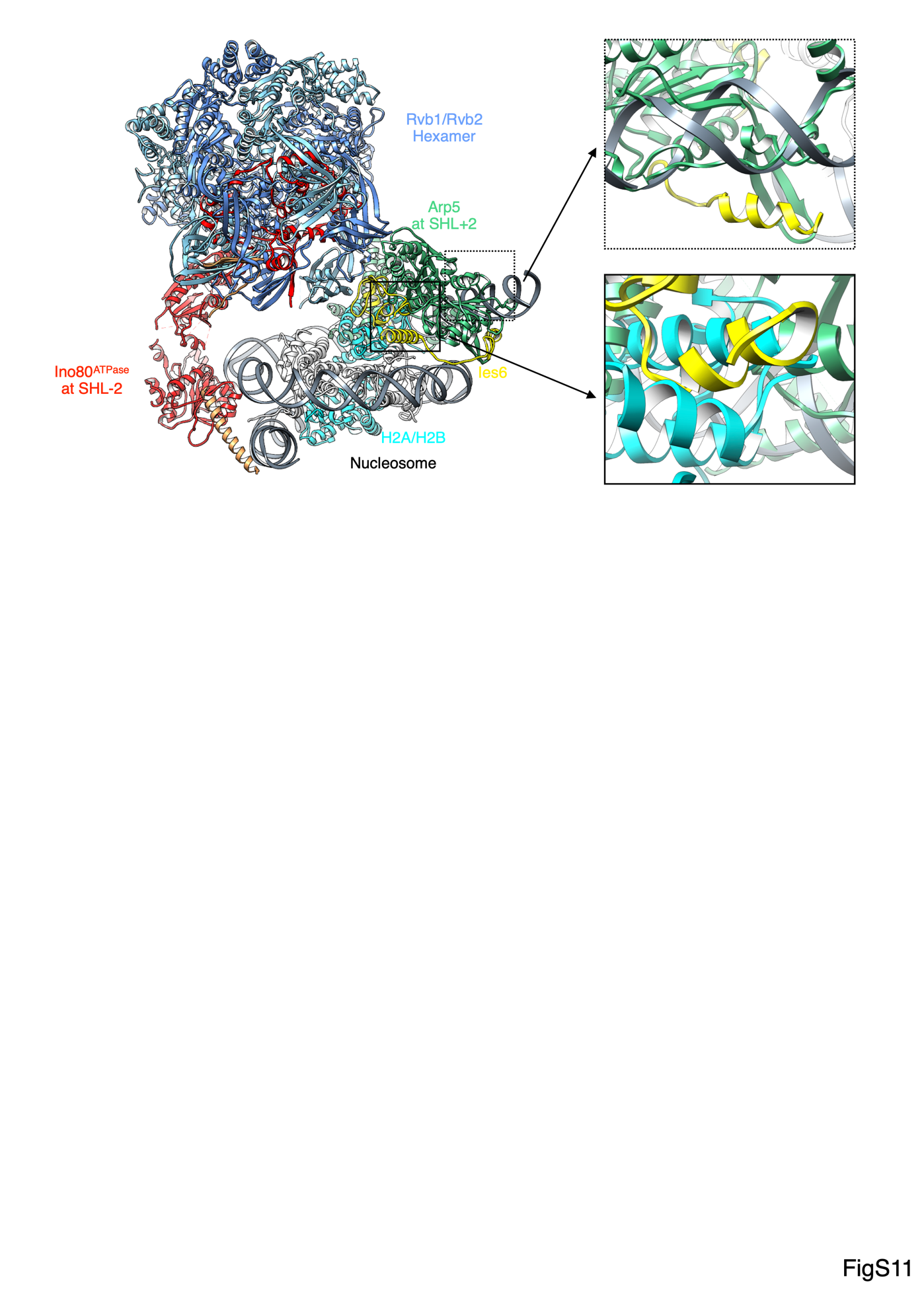
**

**Fig. S11. Modeling INO80 on a nucleosome with Ino80^ATPase^ at SHL-2 results in clashes.**

A hypothetical model of INO80 on a nucleosome made by directly positioning Ino80^ATPase^ at SHL-2 reveals clashes between the Arp5 module and the entry-side H2A-H2B dimer (bottom panel) and between the Arp5 module and DNA that wraps around entry-side H2A-H2B dimer (top panel).

**
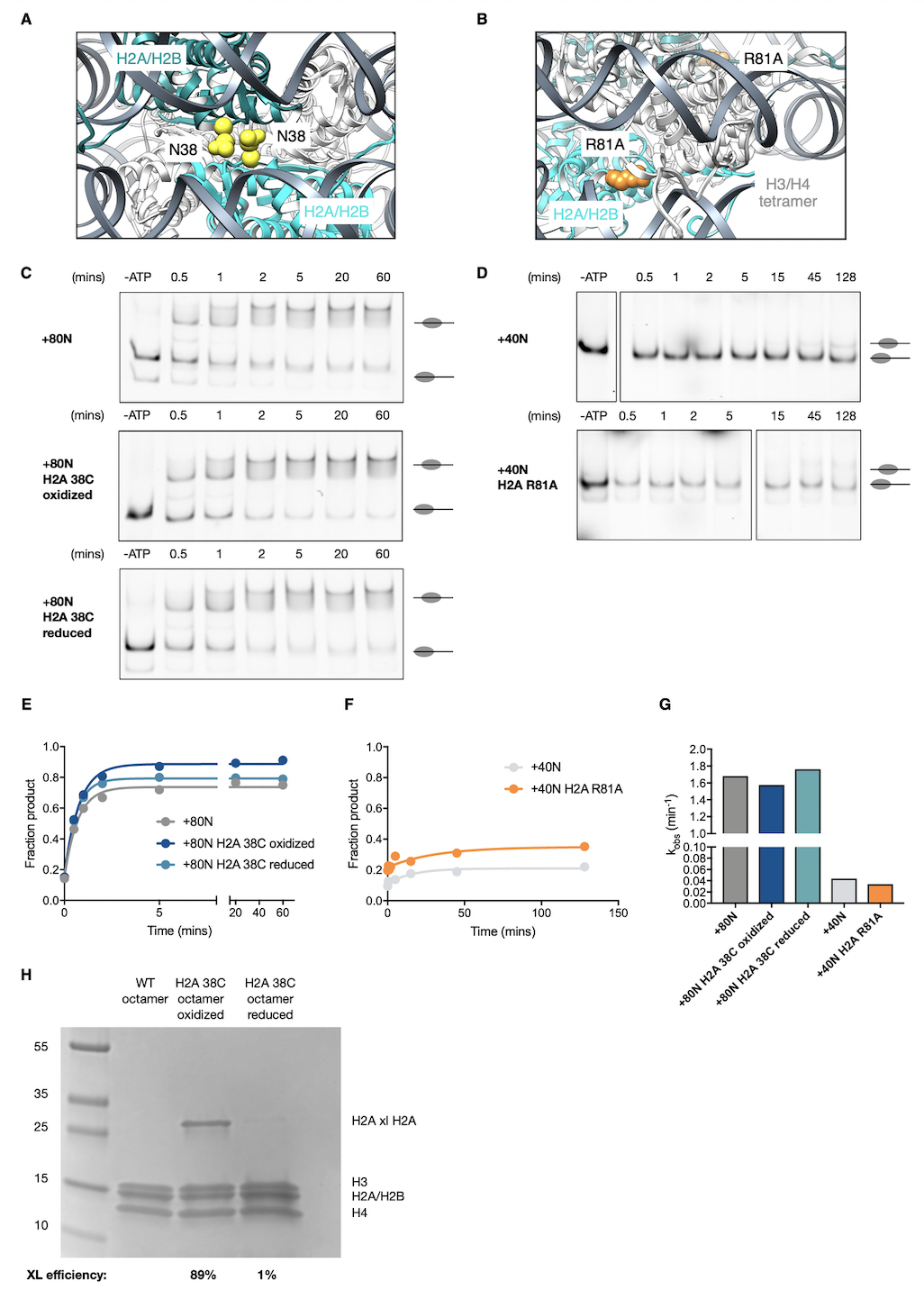
**

**Fig. S12. Crosslinking H2A or destabilizing dimer-tetramer interface does not significantly affect INO80 remodeling activity.**

1. Location of H2A N38C mutation used for crosslinking experiments (PDB: 1KX5).

(B) Location of H2A R81A mutation, which was shown to have destabilizing effects on the nucleosome (PDB: 1KX5).

(C-D) Example gels of native gel-based remodeling assays of (C) WT INO80 on wildtype +80N or +80N containing oxidized or reduced octamers containing H2A 38C; (D) WT INO80 on +40N or +40N containing H2A 81A (+40N R81A).

(E) Example time courses of native gel-based remodeling assays shown in (C).

(F) Example time courses of native gel-based remodeling assays shown in (D).

(G) Average observed rate constants determined from fitting a single-phase exponential decay model to two technical replicates. All assays were performed under single-turnover conditions with saturating enzyme and ATP.

(H) SDS-PAGE gel with wild-type, H2A 38C oxidized, and H2A 38C reduced octamer stained with SYPRO Red. Crosslinking efficiency shown below gel.


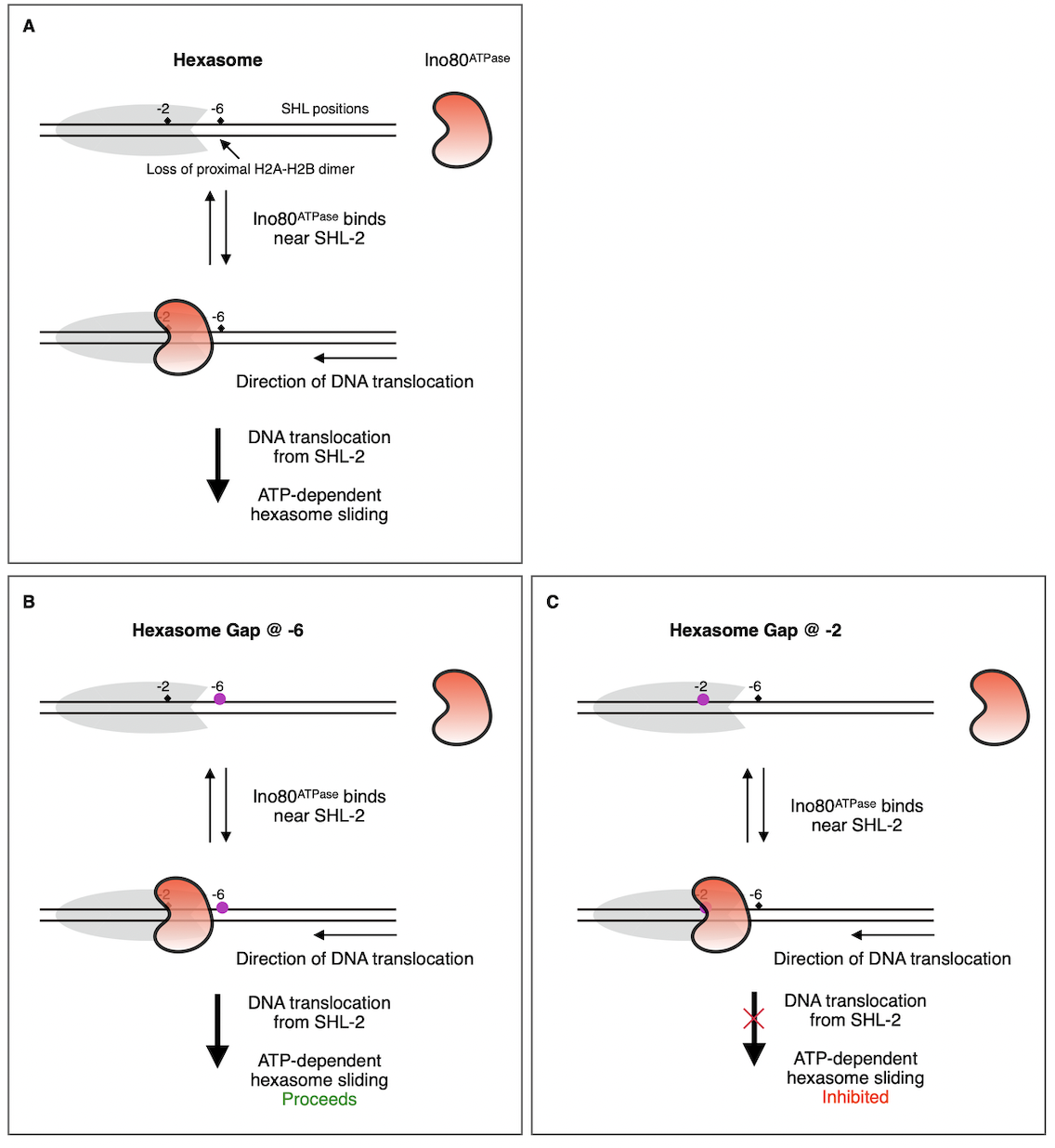


**Fig. S13. Model of INO80-hexasome sliding and predictions of effects of gaps near SHL-6 or -2.**

(A) Model of Ino80^ATPase^ interactions during hexasome sliding by INO80. Only the Ino80^ATPase^ domain of Ino80 is shown for clarity. During sliding of a hexasome, Ino80^ATPase^ first binds near SHL-2. Upon ATP hydrolysis, Ino80^ATPase^ then efficiently translocates DNA from SHL-2 to slide the hexasome.

(B) On a hexasome containing a single base gap near SHL-6, Ino80^ATPase^ binds near SHL-2 and proceeds with hexasome sliding as described in (A).

(C) On a hexasome containing a single base gap near SHL-2, Ino80^ATPase^ binds near SHL-2. The gap near SHL-2 prevents DNA translocation by Ino80^ATPase^ and hexasome sliding is inhibited.


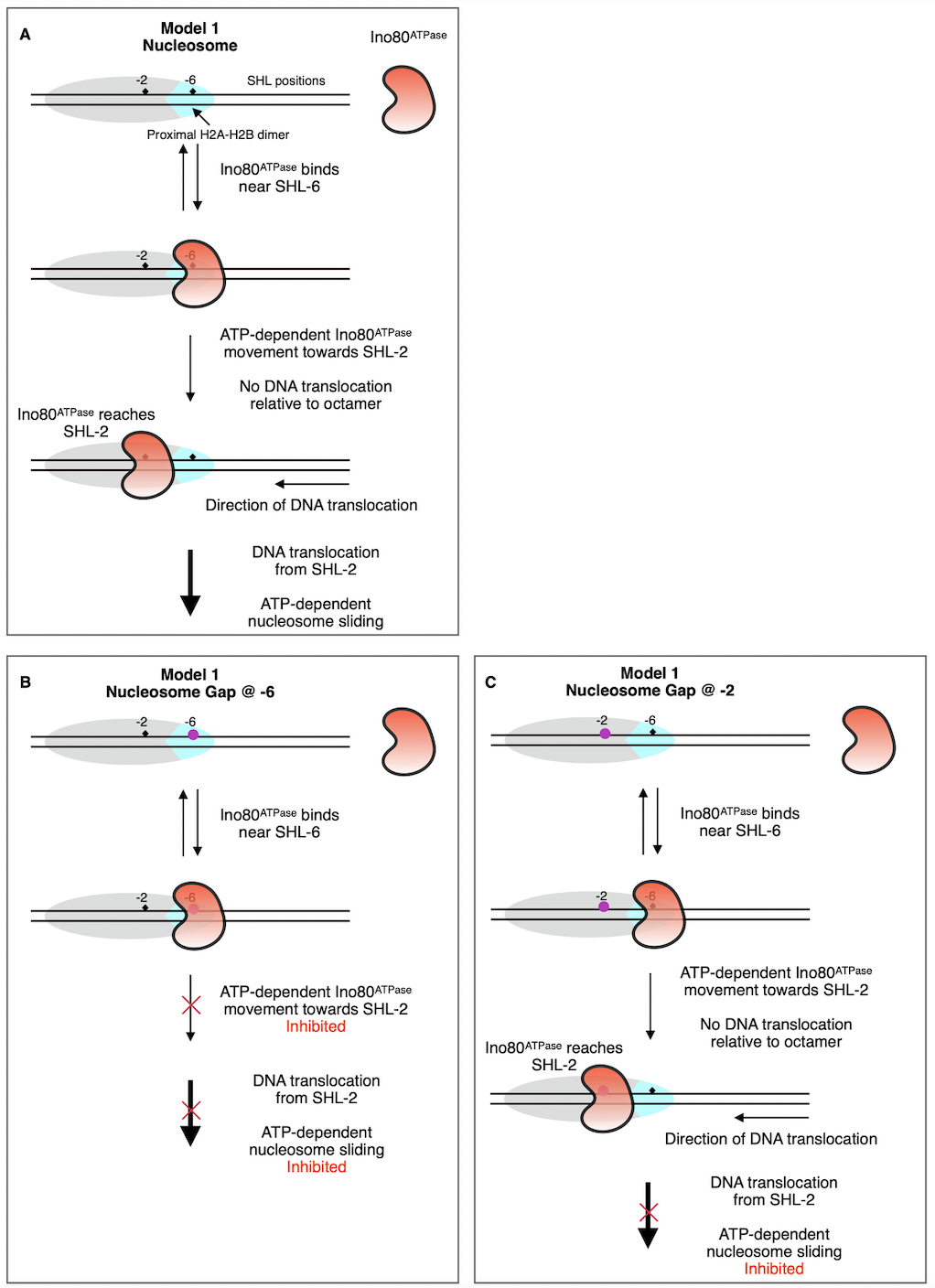


**Fig. S14. Model 1 of INO80-nucleosome sliding and predictions of effects of gaps near SHL-6 or -2.**

(A) Model 1 of Ino80^ATPase^ interactions during nucleosome sliding by INO80. Only the Ino80^ATPase^ domain of Ino80 is shown for clarity. During sliding of a nucleosome, Ino80^ATPase^ first binds near SHL-6. Upon ATP hydrolysis, Ino80^ATPase^ then moves towards SHL-2 without translocating DNA relative to the octamer. Once near SHL-2, Ino80^ATPase^ couples ATP hydrolysis to DNA translocation to slide the nucleosome.

(B) On a nucleosome containing a single base gap near SHL-6, Ino80^ATPase^ binds near SHL-6. The gap near SHL-6 prevents Ino80^ATPase^ from moving towards SHL-2 and therefore downstream nucleosome sliding is inhibited.

(C) On a nucleosome containing a single base gap near SHL-2, Ino80^ATPase^ binds near SHL-6. Upon ATP hydrolysis, Ino80^ATPase^ then moves towards SHL-2 without translocating DNA relative to the octamer. Once the Ino80^ATPase^ is near SHL-2, the gap near SHL-2 prevents DNA translocation by Ino80^ATPase^ and nucleosome sliding is inhibited.


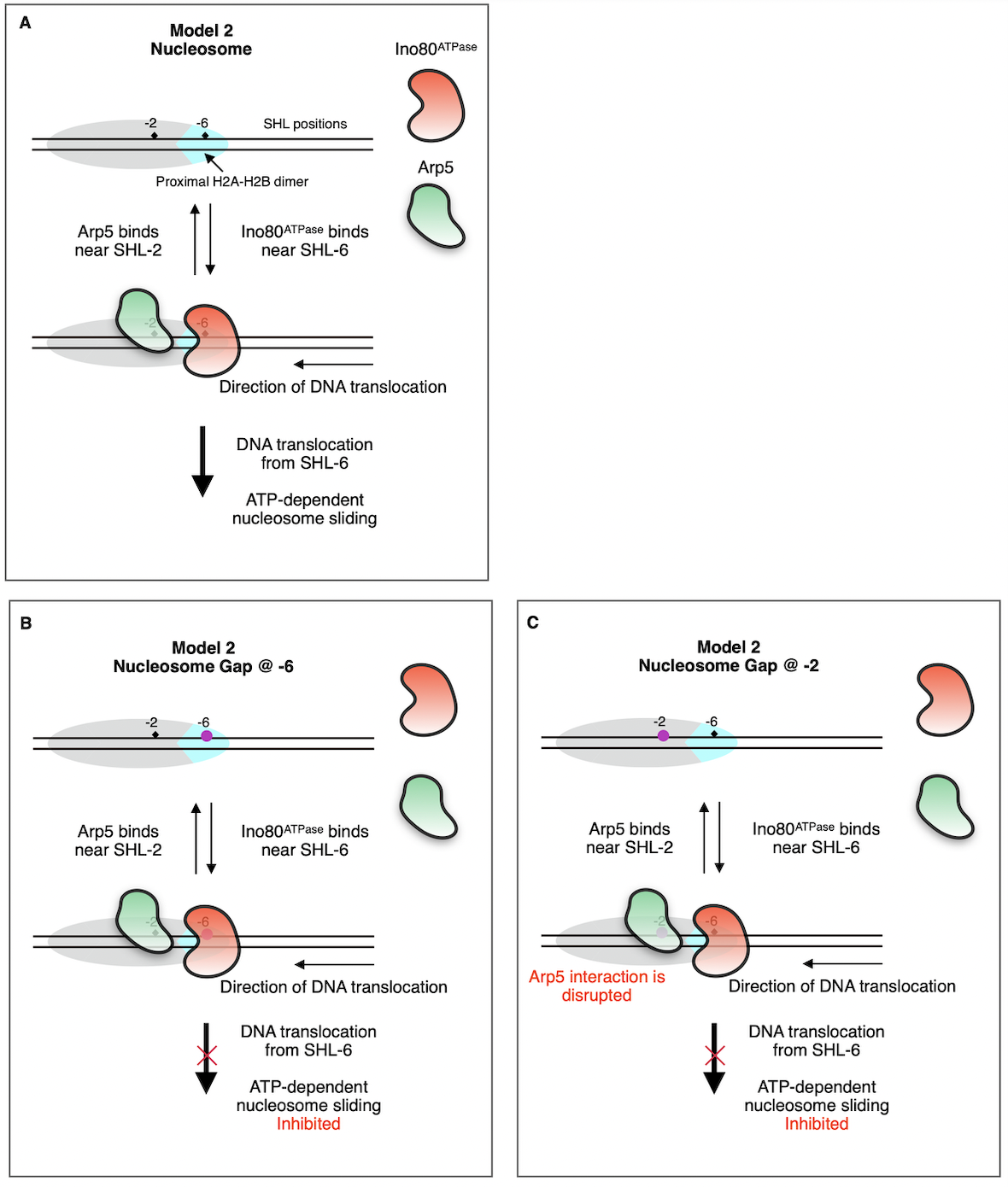


**Fig. S15. Model 2 of INO80-nucleosome sliding and predictions of effects of gaps near SHL-6 or -2.**

(A) Model 2 of Ino80^ATPase^ interactions during nucleosome sliding by INO80. Only the Ino80^ATPase^ domain of Ino80 and the Arp5 subunit are shown for clarity. Ino80^ATPase^ binds near SHL-6 and Arp5 binds near SHL-2. Ino80^ATPase^ translocates DNA from SHL-6 resulting in nucleosome sliding.

(B) On a nucleosome containing a single base gap near SHL-6, Ino80^ATPase^ binds near SHL-6. The gap near SHL-6 prevents DNA translocation by Ino80^ATPase^ and nucleosome sliding is inhibited.

(C) On a nucleosome containing a single base gap near SHL-2, Ino80^ATPase^ binds near SHL-6. The gap near SHL-2 should not inhibit translocation by Ino80^ATPase^ near SHL-6, however, it may inhibit sliding of a nucleosome through disrupting Arp5 module interactions near SHL-2.

**Table S1 Cryo-EM data collection, refinement, and validation statistics of the INO80-Hexasome sample**

| Sample | INO80-Hexasome | | | | | |
| --- | --- | --- | --- | --- | --- | --- |
| Class | Class1 | | Class2 | | Class3 | |
| Region | INO80^core^ | Hexasome | INO80^core^ | Hexasome | INO80^core^ | Hexasome |
| EMDB | 28597 | 28598 | 28599 | 28600 | 28601 | 28602 |
| PDB | 8ETS | 8ETT | 8ETU | 8ETV | 8ETW | 8EU2 |
| **Data collection and Processing** |  | | | | | |
| Microscope | Titan Krios | | | | | |
| Voltage (keV) | 300 | | | | | |
| Camera | Gatan K3 with Gatan Bioquantum energy filter | | | | | |
| Nominal/Calibrated Magnification | 105,000 | | | | | |
| Calibrated Magnification | 119,760 | | | | | |
| Pixel size at detector (Å/pixel) | 0.4175 | | | | | |
| Total electron exposure (e^–^/Å^2^) | 67 | | | | | |
| Exposure rate (e-/pixel/sec) | 16 | | | | | |
| Number of frames | 117 | | | | | |
| Defocus range (μm) | (-0.8) - (-1.8) | | | | | |
| Automation software | SerialEM | | | | | |
| Energy filter slit width (eV) | 20 | | | | | |
| Micrographs used (no.) | 18,991 | | | | | |
| Total extracted/refined particles (no.) | 7,831,514/560,912 | | | | | |
| **Reconstruction** |  | | | | | |
| Final particles | 76,874 | | 130,147 | | 233,675 | |
| Symmetry | C1 | | | | | |
| Resolution (global, Å) | 3.04 | 6.68 | 2.80 | 3.16 | 2.64 | 2.93 |
| Resolution (global, Å)  FSC 0.5 (unmasked/masked)  FSC 0.143 (unmasked/masked) | 3.6/3.4  3.0/3.0 | 9.3/8.6  7.2/6.8 | 3.3/3.1  2.8/2.8 | 4.2/3.9  3.3/3.1 | 3.4/3.1  2.7/2.5 | 3.7/3.4  3.1/2.9 |
| Resolution range (local, Å) | 2.75-3.75 | 5.00-7.00 | 2.50-3.50 | 3.00-4.00 | 2.50-3.50 | 2.75-3.75 |
| Map sharpening *B* factor (Å^2^) | 81.6 | 139.66 | 80.9 | 145.7 | 58.42 | 114.13 |
| 3DFSC Sphericity value | 0.91 | 0.94 | 0.95 | 0.93 | 0.96 | 0.97 |
| **Model composition** |  | | | | | |
| Protein | 3,664 | 524 | 3,664 | 524 | 3,664 | 524 |
| Ligands | 6 | 0 | 6 | 0 | 6 | 0 |
| DNA | 0 | 220 | 0 | 220 | 0 | 220 |
| **Model Refinement** |  | | | | | |
| Refinement package  - real or reciprocal space  - resolution cutoff | Real space  0.143 | Real space  0.143 | Real space  0.143 | Real space  0.143 | Real space  0.143 | Real space  0.143 |
| Model-Map scores  -CC | 0.87 | 0.75 | 0.88 | 0.78 | 0.87 | 0.82 |
| *B* factors (Å^2^) |  | | | | | |
| Protein residues | 147.72 | 309.34 | 131.11 | 90.61 | 58.45 | 75.28 |
| Ligands | 125.68 | N/A | 109.77 | N/A | 43.77 | N/A |
| DNA | N/A | 429.67 | N/A | 130.97 | N/A | 118.55 |
| R.m.s. deviations from ideal values |  | | | | | |
| Bond lengths (Å) | 0.005 | 0.006 | 0.006 | 0.006 | 0.006 | 0.006 |
| Bond angles (°) | 1.136 | 1.123 | 1.207 | 1.006 | 1.203 | 0.960 |
| **Validation** |  | | | | | |
| MolProbity score | 1.34 | 1.38 | 1.53 | 1.13 | 1.54 | 1.07 |
| CaBLAM outliers | 2.04 | 0.60 | 2.23 | 1.00 | 2.26 | 1.00 |
| Clashscore | 3.62 | 4.35 | 5.71 | 2.67 | 5.64 | 1.98 |
| Poor rotamers (%) | 0.00 | 0.00 | 0.00 | 0.00 | 0.00 | 0.00 |
| C-beta deviations | 0.00 | 0.00 | 0.06 | 0.00 | 0.06 | 0.00 |
| EMRinger score | 2.13 | N/A | 2.08 | 1.34 | 2.90 | 1.87 |
| Ramachandran plot  Favored (%)  Allowed (%)  Outliers (%) | 96.88  3.12  0.00 | 97.07  2.93  0.00 | 96.63  3.37  0.00 | 97.66  2.34  0.00 | 96.44  3.56  0.00 | 97.46  2.54  0.00 |

**Table S2 Cryo-EM data collection, refinement, and validation statistics of the INO80-Nucleosome sample**

| Sample | INO80-Nucleosome | | | |
| --- | --- | --- | --- | --- |
| Class | Class1 | | Class2 | |
| Region | INO80^core^ | Nucleosome | INO80^core^ | Nucleosome |
| EMDB | 28609 | 28612 | 28613 | 28614 |
| PDB | 8EU9 | 8EUE | 8EUF | 8EUJ |
| **Data collection and Processing** |  | | | |
| Microscope | Titan Krios | | | |
| Voltage (keV) | 300 | | | |
| Camera | Gatan K3 with Gatan Bioquantum energy filter | | | |
| Nominal Magnification | 105,000 | | | |
| Calibrated Magnification | 117232 | | | |
| Pixel size at detector (Å/pixel) | 0.417 | | | |
| Total electron exposure (e^–^/Å^2^) | 43 | | | |
| Exposure rate (e-/pixel/sec) | 15 | | | |
| Number of frames | 80 | | | |
| Defocus range (μm) | (-0.8) - (-1.8) | | | |
| Automation software | SerialEM | | | |
| Energy filter slit width (eV) | 20 | | | |
| Micrographs used (no.) | 8,653 | | | |
| Total extracted/refined particles (no.) | 3,625,796/132,637 | | | |
| **Reconstruction** |  | | | |
| Final particles | 22,054 | | 44,535 | |
| Symmetry | C1 | | | |
| Resolution (global, Å) | 3.48 | 3.48 | 3.41 | 3.36 |
| Resolution (global, Å)  FSC 0.5 (unmasked/masked)  FSC 0.143 (unmasked/masked) | 4.1/3.8  3.5/3.4 | 6.1/4.3  3.7/3.5 | 4.0/3.8  3.4/3.4 | 6.5/4.1  3.7/3.4 |
| Resolution range (local, Å) | 3.25-4.25 | 3.00-4.00 | 3.25-4.25 | 3.00-4.00 |
| Map sharpening *B* factor (Å^2^) | 57.9 | 92.4 | 57.7 | 35.9 |
| 3DFSC Sphericity value | 0.94 | 0.91 | 0.93 | 0.95 |
| **Model composition** |  | | | |
| Protein | 3,660 | 751 | 3,664 | 747 |
| Ligands | 6 | 0 | 6 | 0 |
| DNA | 0 | 294 | 0 | 294 |
| **Model Refinement** |  | | | |
| Refinement package  - real or reciprocal space  - resolution cutoff | Real space  0.143 | Real space  0.143 | Real space  0.143 | Real space  0.143 |
| Model-Map scores  -CC | 0.85 | 0.80 | 0.84 | 0.76 |
| *B* factors (Å^2^) |  | | | |
| Protein residues | 158.73 | 117.30 | 127.57 | 120.31 |
| Ligands | 128.40 | N/A | 104.07 | N/A |
| DNA | N/A | 192.80 | N/A | 196.19 |
| R.m.s. deviations from ideal values |  | | | |
| Bond lengths (Å) | 0.007 | 0.007 | 0.007 | 0.006 |
| Bond angles (°) | 1.278 | 1.331 | 1.265 | 1.321 |
| **Validation** |  | | | |
| MolProbity score | 1.57 | 1.58 | 1.59 | 1.39 |
| CaBLAM outliers | 2.35 | 1.11 | 2.40 | 1.26 |
| Clashscore | 5.93 | 5.91 | 5.84 | 5.14 |
| Poor rotamers (%) | 0.00 | 0.00 | 0.00 | 0.00 |
| C-beta deviations | 0.00 | 0.00 | 0.03 | 0.00 |
| EMRinger score | 1.51 | 0.64 | 1.57 | 0.82 |
| Ramachandran plot  Favored (%)  Allowed (%)  Outliers (%) | 96.38  3.62  0.00 | 96.19  3.81  0.00 | 96.03  3.97  0.00 | 97.40  2.60  0.00 |
